# A subset of dopamine receptor-expressing neurons in the nucleus accumbens controls feeding and energy homeostasis

**DOI:** 10.1038/s42255-024-01100-0

**Published:** 2024-08-15

**Authors:** Yiqiong Liu, Ying Wang, Zheng-dong Zhao, Guoguang Xie, Chao Zhang, Renchao Chen, Yi Zhang

**Affiliations:** 1 Howard Hughes Medical Institute, Boston Children’s Hospital, Boston, MA USA; 2 Program in Cellular and Molecular Medicine, Boston Children’s Hospital, Boston, MA USA; 3 Division of Hematology/Oncology, Department of Pediatrics, Boston Children’s Hospital, Boston, MA USA; 4 Department of Genetics, Harvard Medical School, Boston, MA USA; 5 Harvard Stem Cell Institute, Boston, MA USA

**Keywords:** Feeding behaviour, Neural circuits, Neuroendocrinology, Feeding behaviour

## Abstract

Orchestrating complex behaviors, such as approaching and consuming food, is critical for survival. In addition to hypothalamus neuronal circuits, the nucleus accumbens (NAc) also controls appetite and satiety. However, specific neuronal subtypes of the NAc that are involved and how the humoral and neuronal signals coordinate to regulate feeding remain incompletely understood. Here we decipher the spatial diversity of neuron subtypes of the NAc shell (NAcSh) and define a dopamine receptor D1-expressing and *Serpinb2-*expressing subtype controlling food consumption in male mice. Chemogenetics and optogenetics-mediated regulation of *Serpinb2*^+^ neurons bidirectionally regulate food seeking and consumption specifically. Circuitry stimulation reveals that the NAcSh^Serpinb2^→LH^LepR^ projection controls refeeding and can overcome leptin-mediated feeding suppression. Furthermore, NAcSh *Serpinb2*^+^ neuron ablation reduces food intake and upregulates energy expenditure, resulting in reduced bodyweight gain. Our study reveals a neural circuit consisting of a molecularly distinct neuronal subtype that bidirectionally regulates energy homeostasis, providing a potential therapeutic target for eating disorders.

## Abstract (web summary)

The work deciphers the spatial diversity of neuronal subtypes of the nucleus accumbens shell and identifies a subset of dopamine receptor-expressing neurons marked by *Serpinb2* that control food seeking and consumption.

## Main

Feeding is a complicated motivational and emotional behavior required for survival and is known for its extraordinary ability to adapt in response to environmental changes^1,2^. The obesity epidemic coupled with an increased population with eating disorders, such as binge eating and anorexia, underscores the urgent need for understanding the mechanisms controlling feeding behavior^3,4^.

Maintaining the balance of energy homeostasis is important for health and survival. The central nervous system has an important role in regulating this balance by coordinated activity of neuronal circuits of corticolimbic entities such as the brain executive^5^ and reward systems^6,7^, and the hypothalamus and brainstem autonomic circuits^8–10^, which control a wide range of physiological processes affecting energy intake and expenditure^11^. The systems and circuits that regulate food intake and energy expenditure are not only sensitive to endogenous homeostatic signals on the status of energy reserves but also to the organoleptic properties of food and emotional factors^12^.

The hypothalamus, with a highly heterogeneous neuronal composition^13^, has a critical role in controlling feeding behavior^14,15^. The core of the hypothalamic control system in the arcuate nucleus (Arc) comprises two neuronal populations: Agouti-related peptide neurons and Pro-opiomelanocortin neurons. These two types of neurons exert almost opposite functions in regulating feeding, with feeding-related hormones such as ghrelin and leptin coordinately mediating the sensations of appetite and satiety, leading to behavioral response^16,17^. Leptin performs an anorexic function by acting on leptin receptors (LepR) in the hypothalamus^18–20^. In addition to the Arc, LepR is also highly expressed in the lateral hypothalamus (LH)^21^, which receives neural inputs from the Arc and dorsomedial nucleus, making it a key brain region for feeding behavior regulation. However, specific inputs outside of the hypothalamus regulating LH^LepR^ neurons in the context of feeding are not known.

Food is naturally rewarding and typically acts on the reward pathways in the brain. The NAc is a key component of the basal ganglion circuitry, which integrates information from cortical and limbic regions to direct feeding behaviors^22^. It has been shown that the pleasure of food consumption is connected with the rostral dorsomedial part of the NAcSh^23^, while the motivation to eat or incentive salience is connected with both shell and core regions^24^. In recent years, several studies have analyzed the role of the mediodorsal NAcSh in feeding^22,25,26^ and revealed that activation of the dopamine receptor 1 expressing medium spiny neurons (D1-MSNs)^27,28^ projecting to the LH^23,26^ or ventral tegmental area (VTA)^29^ stops ongoing food consumption. However, other studies have shown that the activity of D1-MSNs was enhanced during the appetitive phase^30^ as well as during consumption^31^. Although temporally distinct phases of feeding behavior, such as food seeking, evaluation and consumption could potentially account for such a difference, the heterogeneity of NAc neurons^32^ could underlie the discrepancies considering that the studies might have manipulated different neuron subtypes with opposing functions.

With the application of single-cell RNA sequencing and spatial transcriptome techniques, it is now possible to decipher the neuronal heterogeneity in the brain^33–37^, making the study of neuronal circuits and subtype-specific functions of specific behaviors possible. To identify the NAc neuron subtypes that control feeding behavior, we analyzed a multiplexed error-robust fluorescence in situ hybridization (MERFISH) dataset of NAcSh and identified that the *Serpinb2*-expressing D1-MSNs, one of the four D1-MSNs subtypes located in NAcSh, dictates food seeking, goal-directed motivation and food consumption. In addition, through viral tracing and terminal stimulation, we found that the NAcSh^Serpinb2^→LH^LepR^ neuronal circuit modulates feeding behavior and overcomes leptin-mediated feeding suppression. Furthermore, we showed that ablation of the *Serpinb2*^+^ neurons decreases food consumption and increases energy expenditure, resulting in reduced bodyweight gain in mice. Taken together, our results reveal a molecularly distinct accumbal-to-lateral hypothalamic neural circuit whose activity can modulate leptin-mediated effects in the LH, leading to the control of food consumption and bodyweight gain.

## Results

### *Serpinb2*^+^ neurons are activated in the refeeding process

The NAc is a critical component of the basal ganglion circuitry, which receives and integrates information from cortical and limbic regions for actions. Previous studies have linked feeding behavior to the NAcSh^22,23,26,29,30,38^. D1-MSNs, but not D2-MSNs, provide the dominant source of accumbal inhibition to the LH and regulate complex feeding behaviors through LH GABA neurons^22,30^. Given the controversial findings of D1-MSNs in feeding^22,30,31^, studying the specific functions of neuronal subtypes in NAcSh is critical, as the discrepancies could have been caused by manipulation of different neuronal subtypes of NAcSh in the different studies.

**Fig. 1:**
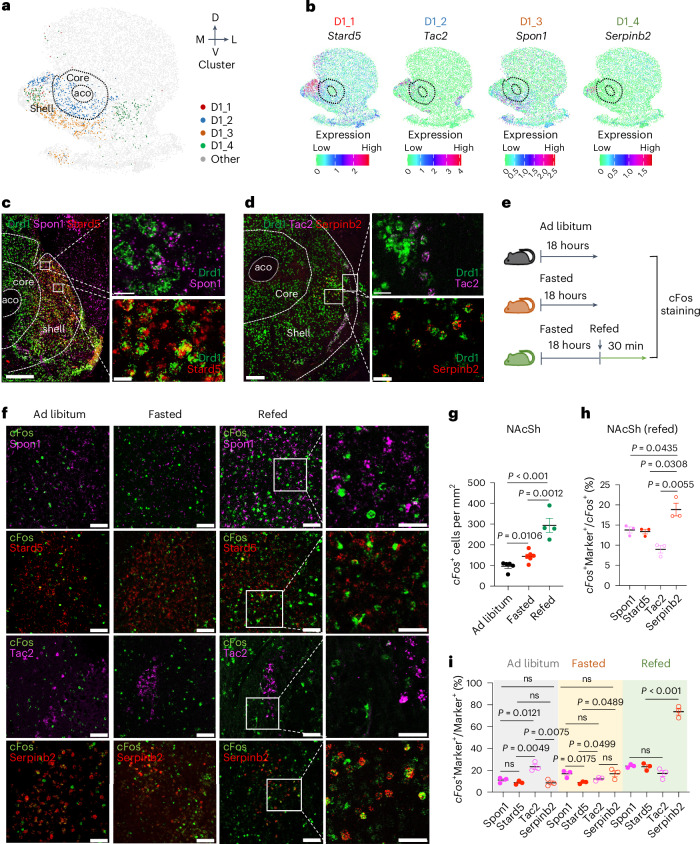
NAcSh *Serpinb2*^+^ neurons are activated in the refeeding process in vivo*.* **a**, Spatial patterns of D1-MSN subtypes in NAcSh. Dotted lines circle the anterior commissure olfactory limb (aco) and NAc core. The dorsal–ventral and medial–lateral axes are indicated. **b**, Spatial expression patterns of *Stard5*, *Tac2*, *Spon1* and *Serpinb2*, determined by integrating single-cell RNA sequencing and MERFISH data using iSpatial. These genes are able to specifically label distinct neuronal subtypes in the medial dorsal NAc shell. Expression level is color-coded. **c**, RNA in situ hybridization showing *Spon1*, *Stard5* and *Drd1* expression in the medial part of the NAcSh. Boxed regions in the left panels are enlarged and shown in the right panels. Scale bars, 500 μm (left), 20 μm (right); *n* = 3 biologically independent experiments. **d**, RNA in situ hybridization showing *Tac2*, *Seprinb2* and *Drd1* expression in the medial part of the NAcSh. Boxed regions in the left panels are enlarged and shown in the right panels. Scale bars, 500 μm (left), 20 μm (right); *n* = 3 biologically independent experiments. **e**, Schematic representation of the experimental design for the ad libitum, fasted and refed groups. **f**, Co-expression of *Spon1, Stard5*, *Tac2* and *Serpinb2* mRNA with *cFos* mRNA in NAc of the ad libitum, fasted and refed states. Representative images showing the colocalization of *cFos* (green), *Spon1* (magenta), *Stard5* (red), *Tac2* (magenta) and *Serpinb2* (red) expressing neurons. Scale bars, 50 μm; *n* = 3 biologically independent experiments. **g**, The average number of *cFos*^+^ neurons in the dorsal medial NAcSh in the ad libitum, fasted and refed states (*n* = 5 sections from three mice of each group; one-way ANOVA with Tukey’s multiple comparison, *F*_2,12_ = 27.82). **h**, The percentages of activated *Spon1, Stard5*, *Tac2* or *Serpinb2*-expressing neurons in the total *cFos*^+^ neurons in dorsal medial NAcSh in the refed state (*n* = 5 sections from three mice of each group; one-way ANOVA with Tukey’s multiple comparisons, *F*_3,8_ = 15.50). **i**, The percentages of activated *Spon1, Stard5*, *Tac2* or *Serpinb2*-expressing neurons in the total *Spon1, Stard5*, *Tac2* or *Serpinb2*-expressing neurons at ad libitum, fasted and refed states (*n* = 5 sections from three mice of each group; one-way ANOVA with Tukey’s multiple comparisons, *F*_11,24_ = 81.44). All error bars represent mean ± s.e.m. Source data

**Extended Data Fig. 1:**
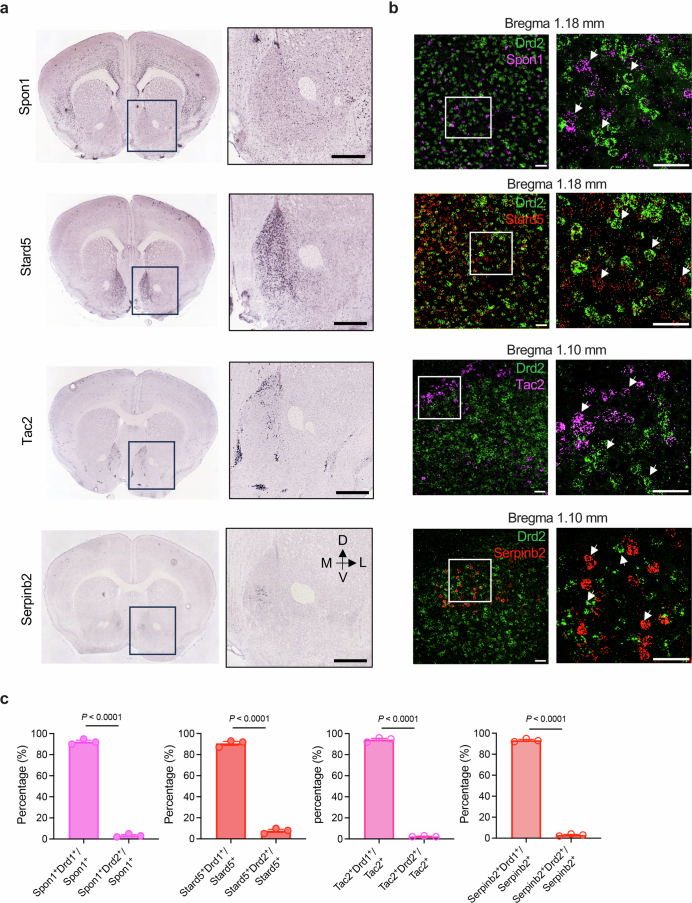
The markers of D1-MSN subtypes in NAcSh do not overlap with Drd2. **a**, ISH images showing the spatial distribution of *Spon1*, *Stard5*, *Tac2* and *Serpinb2* markers of certain D1-MSN subtypes, in mouse NAc. Boxed regions in left panels are enlarged and shown in the right panels. The data are from the Allen Mouse Brain Atlas. Scale bars, 500 µm. **b**, smFISH confirms the expression of D1-MSN subtype-specific markers in the NAcSh do not overlap with Drd2. Boxed regions in left panels are enlarged and shown in the right panels. Three independent experiments were performed with similar results. Scale bar, 50 µm. **c**, The percentage of NAc^Spon1^, NAc^Stard5^, NAc^Tac2^ and NAc^Serpinb2^ cells overlapping with *Drd1*^+^ or *Drd2*^+^ cells in the NAcSh. t_4_ = 52.36 (1st), t_4_ = 35.21 (2nd), t_4_ = 73.2761 (3rd), t_4_ = 85.70 (4th). For **b**,**c**, every fourth of 16-µm brain sections was counted. *n* = 3 mice for all groups. All statistical tests are two-tailed unpaired *t*-tests. Data are mean ± SEM. Source data

To identify the D1 neuronal subtypes located in the NAcSh, we integrated the single-cell and spatial transcriptomic data of NAc^32^ by analyzing 253 selected genes and the coronal sections between 1.94 mm and 0.74 mm from bregma to cover the entire NAcSh region. Clustering analysis of the MERFISH data identified four major cell populations representing D1-MSNs, named D1_1 to D1_4 (Fig. 1a), which are marked by *Stard5*, *Tac2*, *Spon1* and *Serpinb2*, respectively (Fig. 1b). Single-molecule FISH (smFISH) further confirmed that these neuronal subtypes are mainly located in the medial dorsal NAcSh and are predominantly dopamine receptor D1 (*Drd1*^+^) neurons (Fig. 1c,d and Extended Data Fig. 1a–c).

To determine whether any of these D1-MSN subtypes are involved in mediating feeding behavior, we asked whether they are activated in response to feeding by monitoring *cFos* expression under three conditions: ad libitum access to food, after 18 h of fasting and refeeding (Fig. 1e). By counting *cFos*^+^ neurons that co-express *Stard5*, *Tac2*, *Spon1* or *Serpinb2* in the medial dorsal NAcSh of smFISH (Fig. 1f), we found that the D1-MSN subtypes responded differently to the different conditions. Although both fasting and refeeding conditions generally increased neuronal activity compared to the ad libitum condition, as indicated by the increased *cFos*^+^ neuron numbers (Fig. 1g), the *Serpinb2*^+^ neurons exhibited the highest percentage of activation during refeeding, with *Serpinb2*^+^ neurons accounting for 18% of total *cFos*^+^ neurons, and about 70% of *Serpinb2*^+^ neurons are *cFos*^+^ (Fig. 1h,i). By contrast, only 20% of *Stard5*^+^, *Tac2*^+^ and *Spon1*^+^ neurons were activated in the refed condition (Fig. 1i). Collectively, these data indicate that the *Serpinb2*^+^ neurons are the main D1-MSNs subtype in NAcSh that are activated in response to the refeeding process.

### The *Serpinb2*^+^ neurons respond to eating behavior

**Extended Data Fig. 2:**
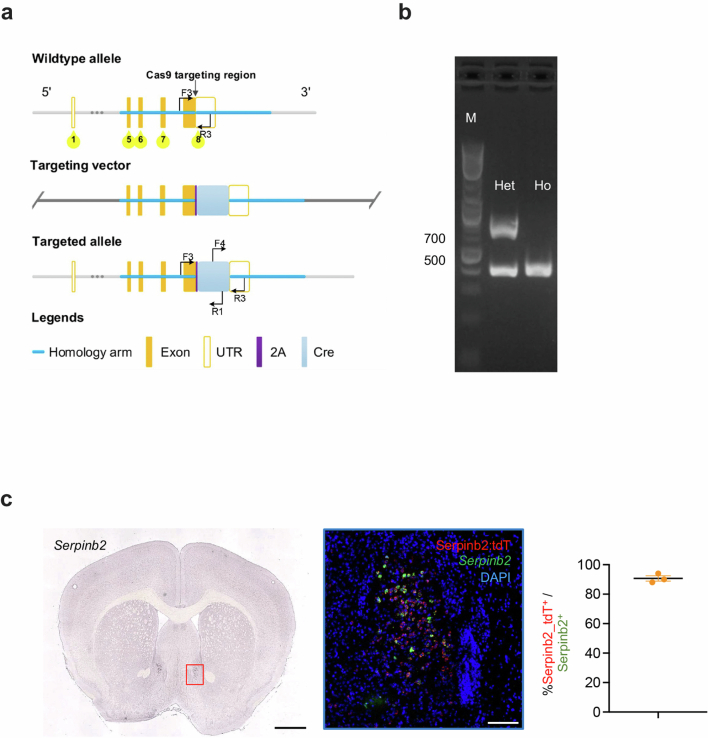
Generation and validation of a *Serpinb2*-Cre mouse line. **a**, Diagrams showing the gene targeting strategy. **b**, Genotyping by PCR. Homozygotes (Ho): 413 bp. Heterozygotes (Het): 413 bp/772 bp. **c**, Left, in situ hybridization (ISH) data of *Serpinb2* from Allen Brain Atlas. Scale bar, 1000 μm. Middle, co-localization of endogenous *Serpinb2* (green), tdTomato (red) and DAPI (blue). Scale bar, 100 μm. Right, quantification of tdTomato^+^ neurons among all *Serpinb2*^+^ neurons. *n* = 3 mice. Data are represented as mean ± SEM. Source data

To study the role of *Serpinb2*^+^ neurons in feeding, we generated a *Serpinb2*-Cre mouse line (Extended Data Fig. 2a,b). We validated this mouse model by crossing with the Ai9 mouse line, which expresses tdTomato (tdT) fluorescence following Cre-mediated recombination, and observed about 90% colocalization of the tdT signal with the endogenous *Serpinb2* mRNA signal, which is consistent with endogenous *Serpinb2* expression in the NAcSh as shown by the Allen Brain Atlas RNA in situ data (Extended Data Fig. 2c). Thus, our *Serpinb2*-Cre mouse line is suitable for studying *Serpinb2*^+^ neurons.

**Fig. 2:**
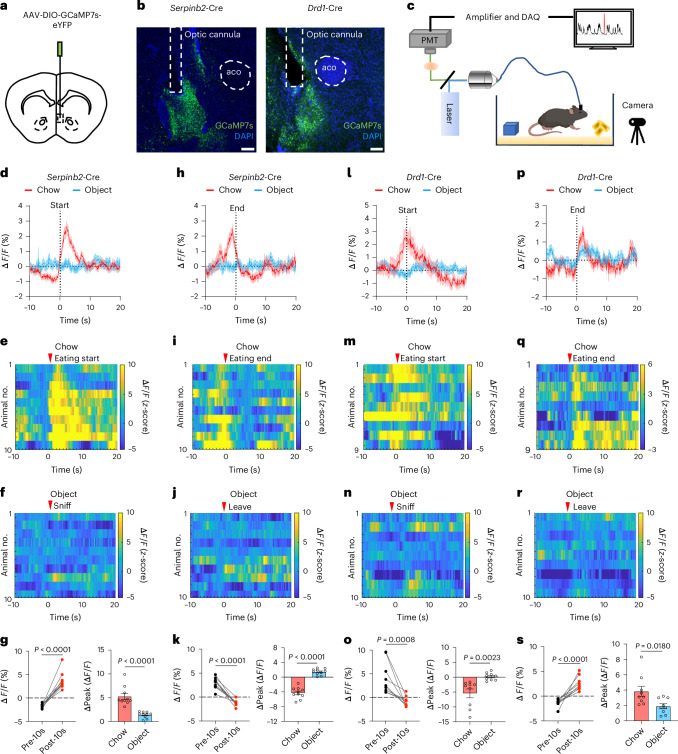
The activity of *Serpinb2*^+^ neurons is increased during feeding. **a**, Diagram of the injection site of AAV-DIO-GCaMP7s virus in NAcSh. **b**, Representative images of GCaMP7s expression and implantation of the optic cannula in *Serpinb2*-Cre mice (left) and *Drd1*-Cre mice (right). The accuracy of cannula insertion was confirmed by histological inspection of each mouse (*Serpinb2*-Cre, *n* = 10 mice; *Drd1*-Cre, *n* = 9 mice). Scale bars, 200 μm. **c**, A schematic setup for recording from NAcSh GCaMP-expressing neurons in a freely behaving mouse. PMT, photomultiplier tubes; DAQ, data acquisition. **d**–**g**, Averaged peri-stimulus histograms of Ca^2+^ signals of *Serpinb2*^+^ neurons. Feeding bouts, <10 s; dashed line, eating start point (**d**). Corresponding heat maps of averaged Δ*F*/*F* (*z*-score) Ca^2+^ responses per animal before and after the onset of eating chow (**e**) and sniffing object (**f**). Averaged Δ*F*/*F* (%) of each individual mouse is represented in the dot plot (**g**, left) and averaged Δpeak Δ*F*/*F* is represented in the bar graph (**g**, right). *n* = 10 mice. Two-tailed, unpaired *t*-test, *t*_18_ = 8.986 (left), *t*_18_ = 5.811 (right). **h**–**k**, Averaged peri-stimulus histograms of Ca^2+^ signals of *Serpinb2*^+^ neurons. Dashed line, eating endpoint (**h**). Corresponding heat maps of averaged Δ*F*/*F* (*z*-score) Ca^2+^ responses per animal before and after the offset of the end of eating chow (**i**) and leaving the object (**j**). Averaged Δ*F*/*F* (%) of each individual mouse is represented in the dot plot (**k**, left), and averaged Δpeak Δ*F*/*F* is represented in the bar graph (**k**, right). *n* = 10 mice. Two-tailed, unpaired *t*-test, *t*_18_ = 10.21 (left), *t*_18_ = 10.38 (right). **l**–**o**, Similar to **d**–**g** but recorded for the *Drd1*-Cre mice. *n* = 9 mice. Two-tailed, unpaired *t*-test, *t*_16_ = 4.086 (left), *t*_16_ = 3.601 (right). **p**–**s**, Similar to **h**–**k** but recorded for the *Drd1*-Cre mice. *n* = 9 mice. Two-tailed, unpaired *t*-test, *t*_16_ = 7.135 (left), *t*_16_ = 2.634 (right). Data are represented as mean ± s.e.m. The graphic of the mouse in **c** was created with BioRender.com. Source data

**Extended Data Fig. 3:**
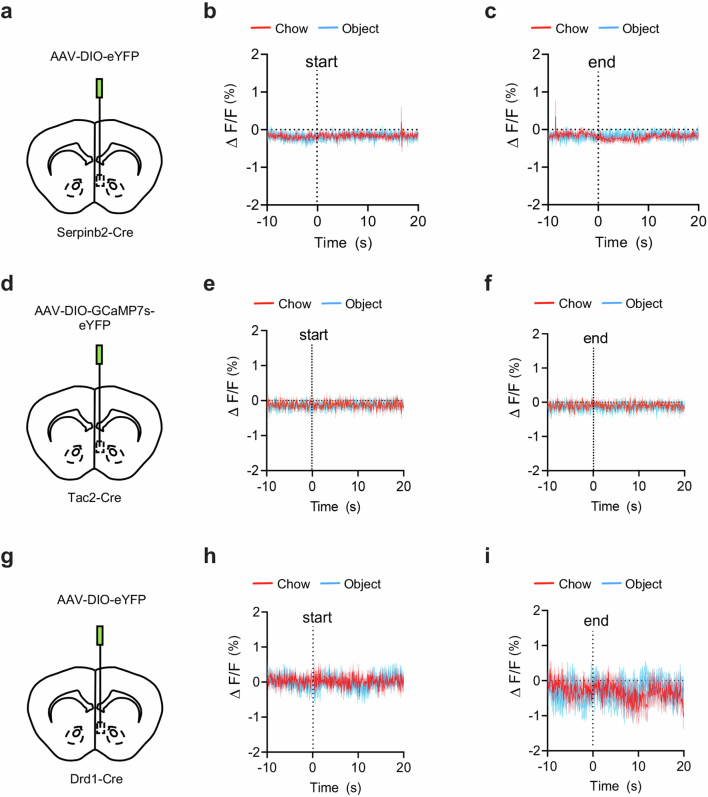
*Tac2*^+^ neurons do not respond to feeding behavior. **a–c**, The injection site of the AAV-DIO-eYFP virus in NAcSh of *Serpinb2*-Cre mice as negative control (**a**). Average peri-stimulus histograms of Ca^2+^ signals of *Serpinb2*^+^ neurons. Dash line, eating start point (**b**). Average peri-stimulus histograms of Ca^2+^ signals of *Serpinb2*^+^ neurons. Dash line, eating end point (**c**). n = 5 mice. **d–f**, Similar as **a–c** but with AAV-DIO-GCaMP7s virus into *Tac2*-Cre mice NAcSh. The Ca^2+^ signal recording is the same as in **b,c**. n = 5 mice. **g–i**, Similar as **a–c** but in *Drd1*-Cre mice. n = 4 mice. Source data

To determine whether *Serpinb2*^+^ neurons are indeed involved in regulating feeding behavior, we used fiber photometry to monitor *Serpinb2*^+^ neuronal activity of freely moving mice during food seeking and consumption. To this end, a Cre-dependent AAV vector expressing the calcium reporter GCaMP7s was delivered to the NAcSh of the *Serpinb2*-Cre mice by stereotaxic injection (Fig. 2a) while AAV expressing eYFP was used as a negative control (Extended Data Fig. 3a), followed by the implantation of an optic cannula above the NAcSh (Fig. 2b). In parallel, we implanted cannula to the *Tac2*-Cre and *Drd1*-Cre mice to monitor the NAcSh *Tac2*^+^ neurons and the total NAcSh *Drd1*^+^ neuron activities during feeding (Extended Data Figs. 3d and 2b). We performed fluorescence recordings during the feeding process in the home cage 3 weeks after the surgeries (Fig. 2c). We found that the Ca^2^^+^ signals of the *Serpinb2*^+^ neurons increased immediately when eating began and decreased when eating finished, whereas there was no obvious change in activity during interaction with a non-food object (Fig. 2d–f,h–j). To quantify the *Serpinb2*^+^ neuronal activity at different events, we averaged the calcium signal of each animal and observed that the peak average signals were significantly increased after the start of eating (Fig. 2g, left) and that these signals were much stronger than those that occurred when sniffing a non-food object (Fig. 2g, right). Consistently, the signals were significantly decreased after the end of eating (Fig. 2k, left) and the change was stronger than occurred when leaving the object (Fig. 2k, right). These results indicate that *Serpinb2*^+^ neuronal activity increases when eating starts and decreases when eating ends. By contrast, we did not capture any obvious activity changes of the *Tac2*-Cre mice during feeding or interacting with the object (Extended Data Fig. 3d–f), indicating that the *Tac2*^+^ neuronal subtype may not be involved in regulating feeding behavior.

We further examined the involvement of the NAcSh *Drd1*^*+*^ neurons in feeding by performing the experiments with the *Drd1*-Cre mice and observed that the Ca^2+^ signals increased before the start of eating and gradually decreased during eating, whereas no obvious change was observed while sniffing the object (Fig. 2l–n). Interestingly, we found that the Ca^2^^+^ signals increased concomitantly at the end of eating, whereas there was no obvious change when leaving the object (Fig. 2p–r). To quantify the Ca^2^^+^ signals of *Drd1*^+^ neurons, we averaged the calcium signal of each animal and found that the peak average signals significantly decreased after the start of eating and significantly increased after the end of eating (Fig. 2o, left, and Fig. 2s, left), and these peaks were much stronger than when the mice were interacting with an object (Fig. 2o, right, and Fig. 2s, right). These results indicate that *Drd1*^+^ neuronal activity increases when approaching the chow, decreases during eating and increases again after the end of eating. As expected, no obvious Ca^2+^ signal changes were detected for the eYFP-injected *Serpinb2*-Cre or *Drd1*-Cre mice when the mice were eating or interacting with an object (Extended Data Fig. 3b,c,h,i).

Collectively, these results indicate that the *Serpinb2*^+^ neurons are activated during eating and the *Tac2*^*+*^ neurons do not respond to the feeding behavior. This differs from the *Drd1*^+^ neurons in NAcSh, which are activated when approaching food and at the end of eating but are inactivated during eating.

### *Serpinb2*^+^ neurons bidirectionally regulate food intake

**Fig. 3:**
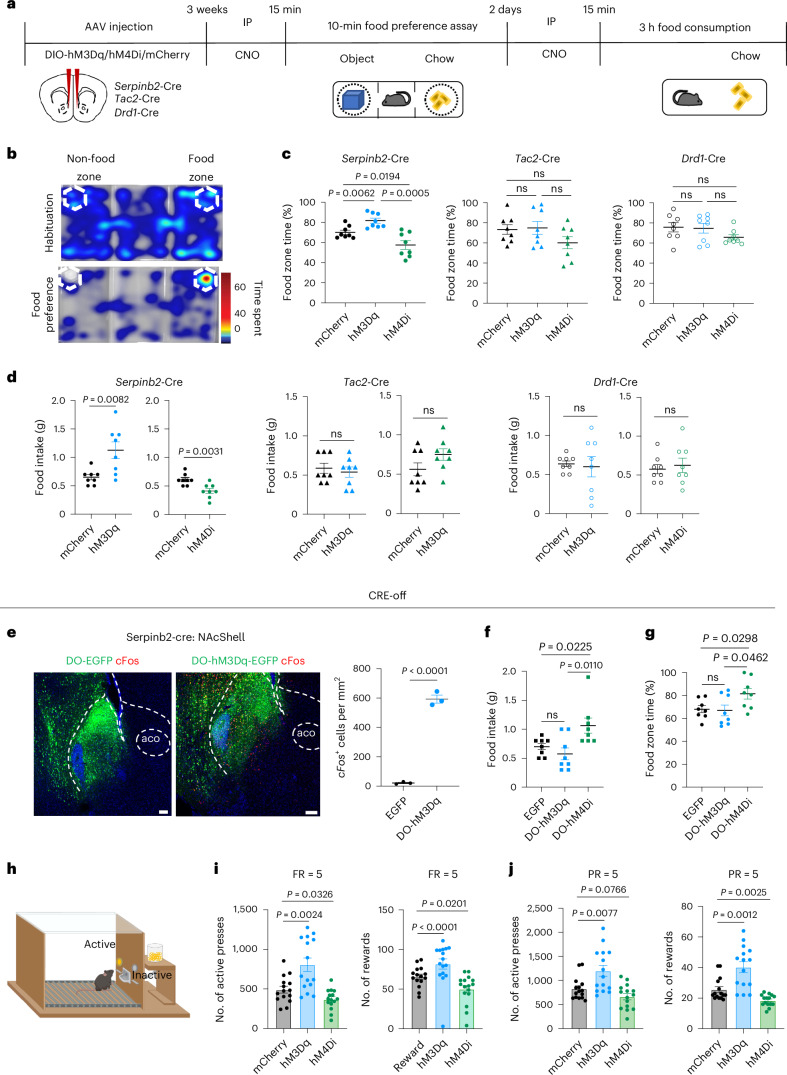
*Serpinb2*^+^ neurons bidirectionally regulate food seeking and intake. **a**, Experimental scheme of the food approach and food consumption assays. **b**, Heat map encoding the spatial location of a fasted mouse using the free-access feeding paradigm. **c**, Percentage of time that mice spent in the food zone. Chemogenetic activation (hM3Dq) or inhibition (hM4Di) of *Serpinb2*^+^ neurons (left), *Tac2*^+^ neurons (middle) and *Drd1*^+^ neurons (right). *n* = 8 mice per group. One-way ANOVA, *F*_2,21_ = 15.05 (left), *F*_2,21_ = 1.970 (middle), *F*_2,21_ = 1.7 (right). **d**, Total food consumption during the 3 h test. Chemogenetic activation (hM3Dq) or inhibition (hM4Di) of *Serpinb2*^+^, *Tac2*^+^ and *Drd1*^+^ neurons. *n* = 8 mice per group. Two-tailed, unpaired *t*-test, *t*_14_ = 0.5472 (*Serpinb2*-Cre, left), *t*_14_ = 3.552 (*Serpinb2*-Cre, right), *t*_14_ = 0.5472 (*Tac2*-Cre, left), *t*_14_ = 1.703 (*Tac2*-Cre, right), *t*_14_ = 0.278 (*Drd1*-Cre, left), *t*_14_ = 0.4571 (*Drd1*-Cre, right). **e**, DO-EGFP/hM3Dq-EGFP virus expression in NAcSh and number of *cFos*^*+*^ neurons after CNO treatment. One technical replicate of three biological replicates. Two-tailed, unpaired *t*-test, *t*_4_ = 20.84. Scale bars, 100 μm. **f**, Percentage of time that mice spent in the food zone. *n* = 8 mice per group. One-way ANOVA test, *F*_2,21_ = 6.2. **g**, Total food consumption during the 3 h test. *n* = 8 mice per group. One-way ANOVA test, *F*_2,21_ = 3.7. **h**, Illustration of the food operant chamber paradigm. Mice were trained to press the lever to get food; pressing the active lever is followed by the delivery of a food pellet; pressing the inactive lever yields no outcome. The behavioral training includes a habituation phase and a fixed ratio (FR) training phase. Mice received CNO injection (2 mg kg^−1^ for the hM3Dq group and 5 mg kg^−1^ for the hM4Di group) 15 min before they were placed into the operant chamber to start the FR and progressive ratio (PR) tests. **i**, Results of FR5 test. The total number of active lever presses (left) and total number of rewards (right) after chemogenetic manipulations. *n* = 15 mice for each group. One-way ANOVA test, *F*_2,42_ = 15.20 (left), *F*_2,44_ = 10.14 (right). **j**, Results of the PR5 test. The total number of active lever presses (left) and total number of rewards (right) after chemogenetic manipulations. *n* = 15 mice for each group. One-way ANOVA test, *F*_2,42_ = 10.70 (left), *F*_2,44_ = 21.19 (right). Data are represented as mean ± s.e.m. The graphics of the mouse and chamber in **h** were created with BioRender.com. Source data

**Extended Data Fig. 4:**
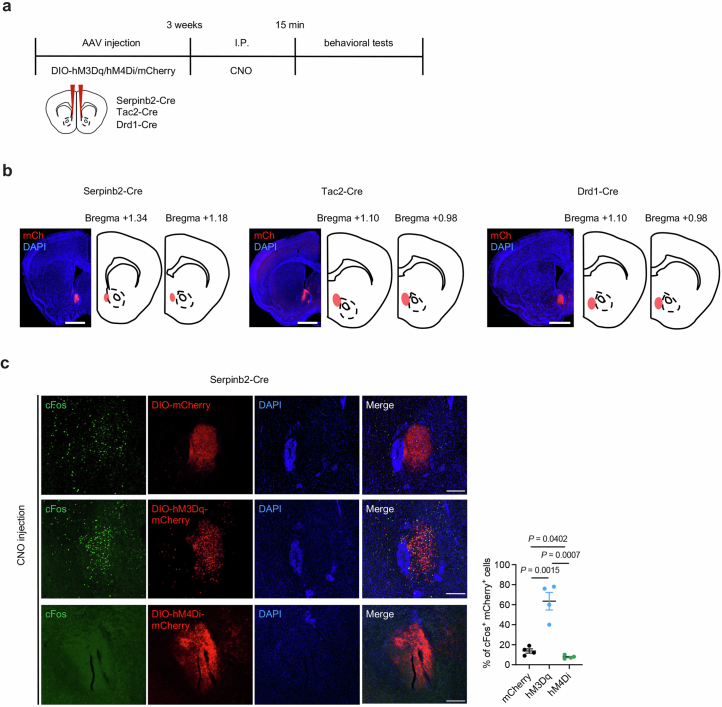
Validation of chemogenetic manipulation. **a**, Experimental scheme of chemogenetic manipulation. **b**, Validation of virus expression in *Serpinb2*-Cre, *Tac2*-Cre and *Drd1*-Cre mice. Scale bar, 500 μm. One technical replicate of three biological replicates. **c**, *cFos* induction after intraperitoneal injection of ligand CNO in mCherry-expressing, hM3Dq-mCherry-expressing and hM4Di-mCherry-expressing mice. The ratio of cFos^+^/mCherry^+^ cells in all mCherry^+^ cells was calculated and shown on the right panel. Scale bar, 100 μm. 4 sections from 4 mice per group. One-way ANOVA test, *F*_2,9_ = 64.56. Data are represented as mean ± SEM. Source data

To determine whether *Serpinb2*^+^ neuronal activity has a causal role in regulating feeding behavior, we examined whether feeding behavior can be influenced by selectively manipulating the *Serpinb2*^+^ neuronal activity. To this end, we injected the Cre-dependent chemogenetic activation vector AAV-DIO-hM3Dq-mCherry or the inhibitory vector AAV-DIO-hM4Di-mCherry into the NAcSh region, using the AAV-DIO-mCherry vector as a control (Fig. 3a). We also performed parallel experiments using *Tac2*-Cre and *Drd1*-Cre mice (Extended Data Fig. 4a).

After confirming the virus expression pattern (Extended Data Fig. 4b) and the efficiency of neuronal activation or inhibition with *cFos* expression (Extended Data Fig. 4c), we then tested whether activation or inhibition of the *Serpinb2*^+^ neurons affects feeding and reward-related behaviors (Fig. 3a). In the food approach assay, during which the mice have free access to three chambers, we analyzed the time the mice spent in the food zone (Fig. 3b). As expected, mice from the control group spent significantly more time in the food zone than in the object zone. Importantly, activation (hM3Dq) or inhibition (hM4Di) of the *Serpinb2*^+^ neurons, respectively, increased or decreased the time that the mice spent in the food zone compared to that of the control mice (Fig. 3c, left). By contrast, similar manipulations of the *Tac2*^+^ or *Drd1*^+^ neurons did not significantly alter the time the mice spent in the food zone compared to that of the control mice (Fig. 3c middle, right). Next, we measured food consumption in the home cage and found that activation of the *Serpinb2*^+^ neurons increased food consumption, while inhibition decreased food consumption (Fig. 3d, left). Similar manipulations performed on *Tac2*-Cre or *Drd1*-Cre mice did not affect food consumption (Fig. 3d middle, right).

Given that our Ca^2+^ imaging results suggested that *Serpinb2*^+^ neuronal activity is positively associated with the start of eating and that NAcSh *Drd1*^+^ neuronal activity decreases upon eating, we reasoned that *Serpinb2*^−^ and *Serpinb2*^+^ neurons play opposite roles in regulating feeding. To test this hypothesis, we used a Cre-off virus (double-floxed orientation, DO) to label all neurons that do not express Cre recombinase and examined the *Serpinb2*^−^ neurons in regulating feeding. To this end, we injected *Serpinb2*-Cre mice with DO-hM3Dq-EGFP/hM4Di-EGFP for activation or inactivation with DO-EGFP serving as a control. Virus expression pattern and activation effect were confirmed by immunostaining (Fig. 3e). We then performed the food approach and food consumption tests on the Cre-off virus-injected mice. Inactivation of *Serpinb2*^−^ neurons induced a significant increase in the time spent in the food zone with a concomitant increase in food consumption (Fig. 3f,g), which is opposite to the manipulation of the *Serpinb2*^+^ neurons (Fig. 3c,d). These results indicate that *Serpinb2*^−^ neurons in NAcSh negatively regulate feeding behavior, counteracting the effects of *Serpinb2*^+^ neurons.

Given that the NAcSh functions as a hub, modulating motivations, we next carried out the food operant chamber test to further determine whether the neuronal activity of the *Serpinb2*^+^ neurons has a causal role in regulating food motivation (Fig. 3h). To maintain a similar food motivation status, all mice were food-restricted to reduce body weight to around 90% of their original value. After being trained to operantly respond to chow pellets at a fixed ratio schedule of 1, 3 or 5 pellets, the animals were then tested for lever pressing upon clozapine-N-oxide (CNO)-induced chemogenetic manipulation. For the fixed ratio 5 test, activation of the *Serpinb2*^+^ neurons significantly increased active lever pressing and pellet reward (Fig. 3i), whereas inhibition elicited the opposite effect (Fig. 3i). A similar result was also obtained when a progressive ratio 5 was used for the test (Fig. 3j). Taken together, these results demonstrate that the *Serpinb2*^+^ neurons are involved in bidirectional control of goal-direct food-seeking behavior.

**Extended Data Fig. 5:**
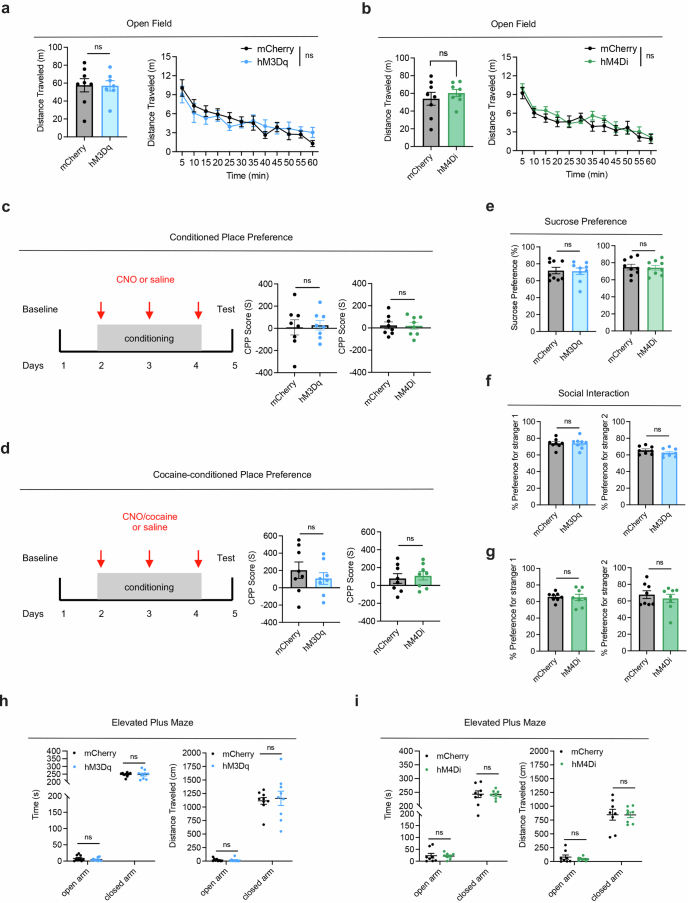
*Serpinb2*^+^ neuronal activity does not affect locomotion, drug seeking, social, anhedonic or anxiety behavior. **a,b**, Open field test for the effect of *Sepinb2*^+^ neuronal activation (hM3Dq) (**a**) or inhibition (hM4Di) (**b**) on the total distance traveled in the 1-hour post-treatment period after chemogenetic manipulation of *Sepinb2*^+^ neurons (left) or the distance traveled in 5-min time bin (right) (**a**, mCherry, 8 mice; hM3Dq, 7 mice; **b**, 8 mice per group). left, two-tailed, unpaired t-test, t_13_ = 0.0626 (**a**), t_14_ = 0.7754 (**b**); right, two-way RM ANOVA, *F*_1,13_ = 0.0039 (**a**), *F*_1,14_ = 0.6012 (**b**). **c**, Left, illustration of the two-chamber CPP paradigm. Right, CPP with chemogenetic activation (hM3Dq) or inhibition (hM4Di) of *Sepinb2*^+^ neurons. CPP scores were calculated by subtracting the time spent in the preconditioning phase from the time spent in the postconditioning phase. n = 8 mice per group. Two-tailed, unpaired t-test, t_14_ = 0.2303 (left), t_14_ = 0.0863 (right). **d**, The same as in panel **c** except conditioned with cocaine. n = 8 mice per group. Two-tailed, unpaired t-test, t_14_ = 0.8196 (left), t_14_ = 0.4226 (right). **e**, Sucrose preference test for the effect of *Sepinb2*^+^ neuronal activation (hM3Dq) (left) or inhibition (hM4Di) (right). n = 9 mice per group. Two-tailed, unpaired t-test, t_16_ = 0.1470 (left), t_16_ = 0.1821 (right). **f,g**, 3 chamber social interaction test for the effect of *Sepinb2*^+^ neuronal activation (hM3Dq) (top) or inhibition (hM4Di) (bottom). n = 8 mice per group. Two-tailed, unpaired t-test, t_14_ = 0.0142 (**f**, left), t_14_ = 1.200 (**f**, right), t_14_ = 0.1427 (**g**, left), t_14_ = 0.6685 (**g**, right), **h,i**, Elevated plus maze test for the effect of *Sepinb2*^+^ neuronal activation (hM3Dq) (**h**) or inhibition (hM4Di) (**i**) on the time spent (left) or distance traveled (right) in open arm and closed arm of the 5-min post-treatment period after chemogenetic manipulation of *Sepinb2*^+^ neurons. n = 8 mice per group. Two-tailed, unpaired t-test, t_16_ = 1.010 (**h**, left, open arm), t_16_ = 0.0556 (**h**, left, closed arm), t_16_ = 0.2663 (**h**, right, open arm), t_16_ = 0.3168 (**h**, right, closed arm). t_14_ = 0.1410 (**i**, left, open arm), t_14_ = 0.1252 (**i**, left, closed arm), t_14_ = 0.8526 (**i**, right, open arm), t_14_ = 0.0247 (**i**, right, closed arm). Data are represented as mean ± SEM. Source data

In addition to food consumption, the NAcSh has been shown to participate in other motivated behaviors, including conditioned reinforcement^39,40^, hedonic reactivity^41^, anxiety^42^ and social play^43^. Thus, we asked whether *Serpinb2*^+^ neurons also regulate these behaviors under the same chemogenetic manipulation. We found that manipulation of *Serpinb2*^+^ neuronal activity did not affect locomotion in an open field test (Extended Data Fig. 5a,b), conditioned place preference (CPP) (Extended Data Fig. 5c), cocaine CPP test (Extended Data Fig. 5d), anhedonia in sucrose preference test (Extended Data Fig. 5e), social interaction (Extended Data Fig. 5f,g) or anxiety in the elevated plus maze test (Extended Data Fig. 5h,i). These results support that *Serpinb2*^+^ neurons are specifically involved in feeding, but not in locomotion, anxiety, social, anhedonia or drug-seeking behaviors.

### *Serpinb2*^+^ neurons mediate food consumption by LH projection

**Fig. 4:**
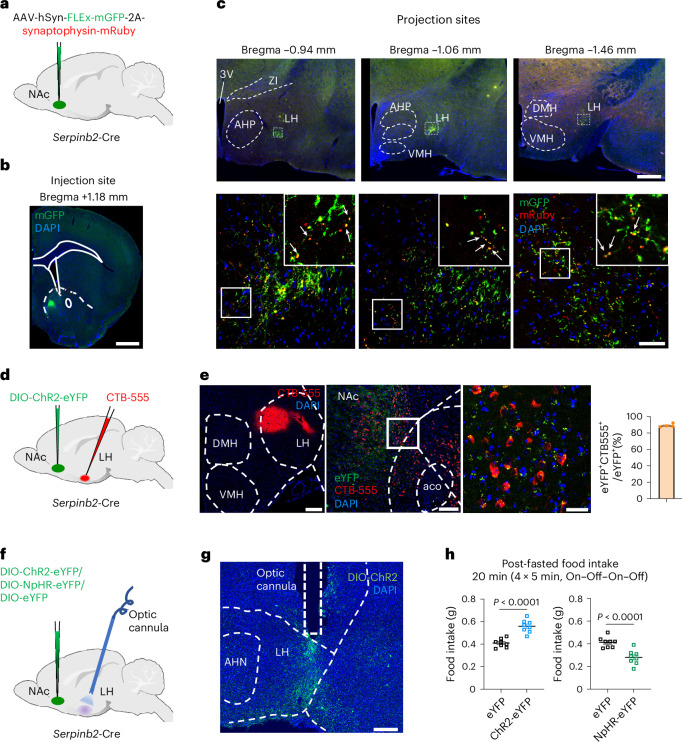
*Serpinb2*^+^ neurons mediate food intake via LH projection. **a**,**b**, Diagram of injection of the NAc *Serpinb2*^+^ neurons with AAV-hSyn-FLEx-mGFP-2A-synaptophysin-mRuby (**a**) and the expression FLEx-mGFP in the NAcSh (**b**). Scale bar, 100 μm. One technical replicate of three biological replicates. **c**, Top, *Serpinb2*^+^ neuron projection to the LH. Scale bar, 500 μm. Bottom, enlarged image of the top square. Square in the right is an enlargement of the left. Arrows indicate the colocalization. Scale bar, 50 μm. **d**, Diagram of retrograde tracing approach. **e**, Left, validation of injection site of CTB-555 in the hypothalamus. Middle, validation of injection site of DIO-ChR2-eYFP in the NAcSh. Right, colocalization of retrograde tracing of LH neurons with CTB-555, with the *Serpinb2*^+^ neurons labeled by AAV-DIO-ChR2-eYFP. Scale bars, 200 μm (left), 100 μm (middle), 30 μm (right). *n* = 4 mice. **f**,**g**, Diagram illustrating the indicated AAV injection into NAc and optic cannulas implantation in the LH area (**f**) and histology validating virus expression and cannula implantation site (**g**). Scale bar, 200 μm. **h**, Optogenetic activation (left) or inhibition (right) of NAc^*Serpinb2*+^→LH neurons, respectively increasing or decreasing food intake. *n* = 8 mice per group. Two-tailed, unpaired *t*-test, *t*_14_ = 6.195 (left), *t*_14_ = 4.773 (right). Data in **h** are presented as mean ± s.e.m. 3V, third ventricle; AHN, anterior hypothalamic nucleus; AHP, anterior hypothalamic area, posterior part; DMH, ventromedial hypothalamic nucleus; HY, hypothalamus; VMH, ventromedial hypothalamic nucleus; ZI, zona incerta. Source data

**Extended Data Fig. 6:**
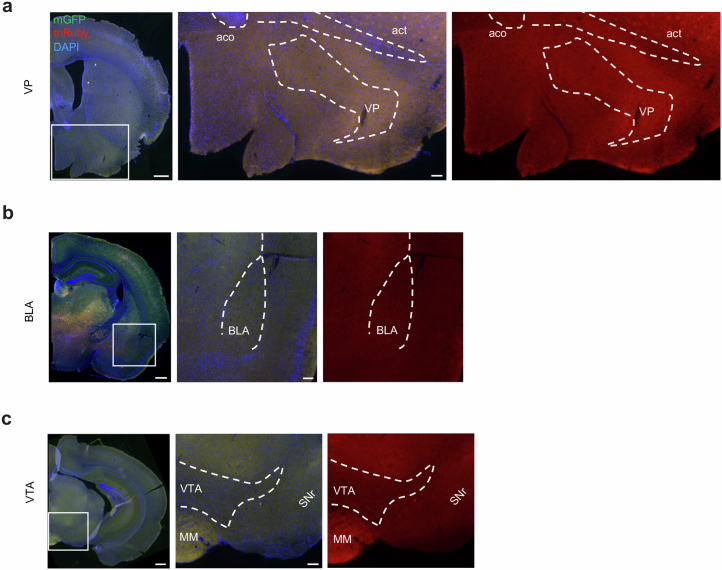
Projection mapping of *Serpinb2*^+^ neurons. **a–c**, No detectable signals of mGFP or mRuby in VP (**a**), BLA (**b**) or VTA regions (**c**). aco, anterior commissure; act, anterior commissure, temporal limb; BLA: Basolateral amygdala; MM, medial mammillary nucleus; SNr, substantia nigra, reticular part. Scale bars, 500 μm (left), 100 μm (middle).

Thus far, we have demonstrated the critical role of *Serpinb2*^+^ neurons in regulating feeding behaviors. Next, we attempted to reveal the circuit mechanism underlying the functions of *Serpinb2*^+^ neurons in regulating food consumption. Previous studies have indicated that NAcSh D1-MSNs project to multiple brain regions, including the VTA^44^, LH^22^ and ventral pallidum^45^. To determine the projection sites of *Serpinb2*^+^ neurons, we injected Cre-dependent AAVs expressing membrane-bound GFP (mGFP, for labeling axons) and synaptophysin-mRuby, for labeling putative presynaptic sites, into the NAcSh of the *Serpinb2*-Cre mice (Fig. 4a). We analyzed the brain sections 3 weeks after virus injection and observed colocalization of green neuronal terminals and red presynaptic puncta only in the LH (Fig. 4b,c) but not in the ventral pallidum, basolateral amygdala or VTA (Extended Data Fig. 6), indicating that the *Serpinb2*^+^ neurons only project to the LH. To further validate this projection, we injected the retrograde tracer Cholera toxin subunit B (CTB) conjugated with Alexa Fluor 555 (CTB-555)^46^ into the LH region (Fig. 4d) and AAV-DIO-ChR2-eYFP into the NAcSh of *Serpinb2*-Cre mice. Immunostaining showed colocalization of CTB with eYFP in the NAcSh (Fig. 4e), supporting that the NAcSh *Serpinb2*^+^ neurons project to the LH.

To demonstrate that the LH projection of *Serpinb2*^+^ neurons is functionally relevant to feeding, we asked whether optogenetic manipulation of the *Serpinb2*^+^ neuron terminals in the LH can regulate feeding behavior. To this end, AAV-DIO-ChR2-eYFP or AAV-DIO-NpHR-eYFP vectors were injected into the NAcSh of the *Serpinb2*-Cre mice with an optic cannula implanted into their LH region; the AAV-DIO-eYFP vector was used as a control (Fig. 4f,g). After fasting the mice overnight, we activated the *Serpinb2*^+^ neuron terminals in the LH with blue light on–off stimulation (20 Hz, 2 ms pulses). We found that stimulation of *Serpinb2*^+^ neuron terminals in ChR2-expressing mice significantly increased the food intake compared to the control mice that express eYFP (Fig. 4h, left). Conversely, inhibition of the *Serpinb2*^+^ neuron terminal in the LH with yellow light decreased total food intake compared to the control (Fig. 4h, right). Collectively, viral tracing and circuit manipulation demonstrate that the projection from NAcSh *Serpinb2*^*+*^ neurons to the LH is functionally important for feeding behavior.

### *Serpinb2*^+^ neurons form a circuit with LH LepR^+^ GABA^+^ neurons and counterbalance the effects of leptin

**Fig. 5:**
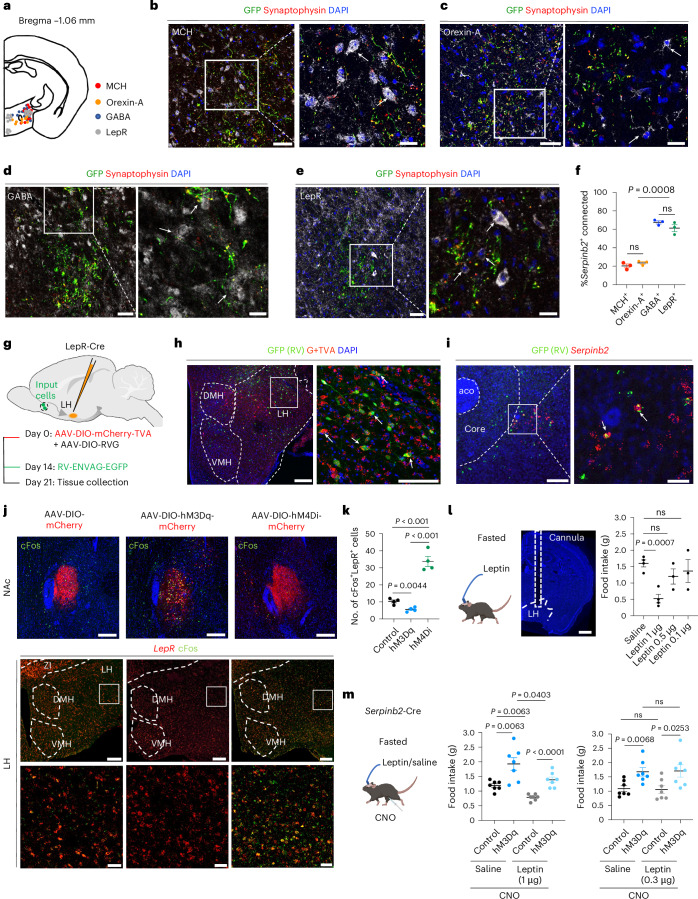
*Serpinb2*^+^ neurons project to LH LepR^+^ GABA^+^ neurons and their activation can counteract the effect of leptin. **a**, Diagram indicating the MCH^+^, orexin-A^+^, GABA^+^ and LepR^+^ neurons in the LH. **b**,**c**, Example images of non-connected MCH^+^ (**b**) and orexin-A^+^ neurons (gray) (**c**) and *Serpinb2*^+^ neuron terminals (green fibers with red puncta) in the LH. Scale bars, 50 μm (left), 20 μm (right). Arrows indicate somas. **d**,**e**, Images showing the colocalization of *Serpinb2*^+^ neuron terminals (green fibers with red puncta) with GABA^+^ (**d**) and LepR^+^ (gray) (**e**) as indicated by arrows. Scale bars, 50 μm (left), 20 μm (right). One technical replicate of three biological replicates. **f**, Quantification of *Serpinb2*^+^ neuron terminal connected cells. Percentage represents eYFP^+^MCH^+^/eYFP^+^DAPI^+^, eYFP^+^orexin-A^+^/eYFP^+^DAPI^+^, eYFP^+^GABA^+^/eYFP^+^DAPI^+^ or eYFP^+^LepR^+^/eYFP^+^DAPI^+^. *n* = 3 sections from three mice. One-way ANOVA test, *F*_3,8_ = 101. **g**, The timeline of monosynaptic retrograde rabies tracing of LH^LepR^ neurons. *n* = 4 biological replicates. **h**, Representative images showing the location of starter LH^LepR^ neurons in a LepR-Cre mouse; mCherry-TVA (red), rabies-GFP (green) and DAPI (blue). Scale bars, 100 µm (left), 50 µm (right). **i**, Representative histological images with cells retrogradely labeled from LH^LepR^ neurons (green) and *Serpinb2*^+^ neurons (red). Scale bars, 200 µm (left), 50 µm (right). **j**, cFos detection in the LH of DREAAD-treated *Serpinb2*-Cre mice. Virus expression in NAcSh (top); mCherry (red), cFos (green) and DAPI (blue). CNO-induced cFos immunofluorescence signals were observed in the LH; bottom panels are enlarged images of the squares above. Scale bars, 100 µm (top), 200 µm (middle) and 50 µm (bottom). **k**, The number of cFos^+^ cells (*n* = 4 sections from four mice). One-way ANOVA test, *F*_2,9_ = 69.51. **l**, Diagram showing cannula implantation in LH for leptin delivery (left, middle). Results of total food consumption in 3 h by fasted mice with different doses of leptin administration (right). Scale bar, 500 μm. One-way ANOVA test, *F*_3,10_ = 6.025. **m**, Same as **k** except food consumption is quantified under different conditions with or without *Serpinb2*^+^ neuron activation in the presence or absence of 1 μg (middle) or 300 ng (right) of leptin delivery. One-way ANOVA test. *F*_3,24_ = 14.72 (left), *F*_3,24_ = 5.285 (right). Data are presented as mean ± s.e.m. The graphics of the mouse and syringe in **l** and **m** were created with BioRender.com. Source data

Having demonstrated the functional importance of the NAcSh-to-LH projection, we next attempted to determine the neuron types in the LH that receive signals from the *Serpinb2*^+^ neurons. The LH is a highly heterogeneous brain region, controlling food intake, energy expenditure and many other physiological functions^47^. Given that the neuropeptides orexin/hypocretin and melanin-concentrating hormone (MCH) are associated with feeding^48,49^ and are mainly expressed in the LH, we first asked whether they are the downstream targets of the NAcSh *Serpinb2*^+^ neurons. To this end, we injected the AAV-hSyn-FLEx-mGFP-2A-synaptophysin-mRuby viruses to the NAcSh of the *Serpinb2*-Cre mice and performed immunostaining of candidate neuropeptides or transmitters on slices covering the LH (Fig. 5a). We found that a small number of MCH-expressing or orexin-expressing neurons in the LH (Fig. 5b,c,f, indicated by arrows) overlapped with *Serpinb2*^+^ terminals (green). Given that GABAergic neurons are the most abundant subtype in the LH and are involved in feeding and leptin-regulated energy homeostasis^13,50,51^, we next checked whether LepR-positive GABAergic neurons^21^ receive projections from NAcSh *Serpinb2*^+^ neurons. After immunostaining with anti-GABA or anti-LepR antibodies, we found that the presynaptic puncta of *Serpinb2*^+^ neuron terminals were located around the LepR^+^ GABA^+^ somas (Fig. 5d–f, arrowheads). To confirm the connection of NAcSh *Serpinb2*^+^ neurons and LH *LepR*^+^ neurons, we performed monosynaptic rabies virus tracing by injecting Cre-dependent AAVs expressing mCherry-TVA and rabies G protein into the LH of LepR-Cre mice. After 2 weeks, Enva-ΔG rabies virus expressing GFP was injected into the same location, which revealed NAc as one of the inputs for LH^LepR+^ neurons (Fig. 5g,h). smFISH further confirmed that the GFP-labeled neurons are *Serpinb2*-expressing neurons (Fig. 5i), which supports that NAcSh *Serpinb2*^+^ neurons directly target LH^LepR+^ neurons. To demonstrate that the NAcSh^Serpinb2+^–LH^LepR+^ circuit is functional, we selectively activated or inhibited *Serpinb2*^+^ neurons and then analyzed the LH^LepR+^ neuronal response. Given that MSNs in NAc are GABAergic neurons, inhibition of *Serpinb2*^+^ neurons would lead to a decreased inhibition effect on the downstream neurons. As expected, we observed increased *cFos* signals, which merged with the LepR signal in the LH (Fig. 5j, bottom right). Importantly, inhibition of *Serpinb2*^+^ neurons significantly increased the *cFos*^+^ neurons in the LH compared to that in the control (mCherry) or activation (hM3Dq) groups (Fig. 5k). Thus, NAcSh^Serpinb2+^ neurons are functionally connected with LH^LepR+^ neurons.

As an adipose-derived hormone, leptin has a central role in regulating energy homeostasis^52–54^. Leptin performs most of its functions, including suppression of food intake, by activating the LepR on central nervous system neurons^55,56^. As the NAcSh *Serpinb2*^+^ neurons project to LepR^+^ GABAergic neurons in the LH (Fig. 5g–k), we anticipated that both leptin and the NAcSh *Serpinb2*^+^ neurons have shared neuron targets and consequently regulate feeding behavior through a converged mechanism. To test for their potential functional interaction in food intake, we implanted a catheter in the LH for leptin delivery (catheter administration) in the *Serpinb2-*Cre mice that were also injected with hM3Dq-mCherry-expressing AAV into the NAcSh so that the NAcSh *Serpinb2*^+^ neurons can be activated by CNO through intraperitoneal injection. After establishing that 1 μg of bilateral intra-LH leptin cannula delivery^57^ can significantly decrease food intake in 3 h relative to the saline control (Fig. 5l), we used 1 μg of leptin for all subsequent tests. Consistent with what we have shown (Fig. 3d), CNO-induced *Serpinb2*^+^ neuron activation increased food intake (Fig. 5m, middle, control vs hM3Dq in saline). Importantly, despite leptin-delivery reduced food intake, the effect of 1 μg of leptin can be at least partly overcome by CNO-induced *Serpinb2*^+^ neuron activation (Fig. 5m, middle, hM3Dq with or without leptin), whereas a lower dose of leptin (300 ng) did not exhibit a similar effect (Fig. 5m, right). These results indicate that the leptin-induced inhibitory effect on food intake can be at least partly overcome by *Serpinb2*^+^ neuron activation.

### Ablation of *Serpinb2*^+^ neurons promotes weight loss and increased metabolic level

**Fig. 6:**
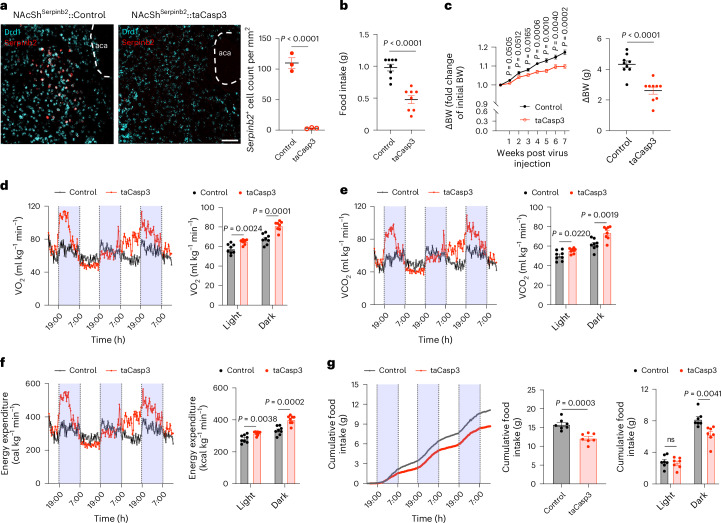
Ablation of *Serpinb2*^+^ neurons decrease bodyweight gain by reducing food consumption and increasing energy expenditure. **a**, FISH and quantification verify *Serpinb2*^+^ neuron ablation after AAV-DIO-taCasp3 injection. Scale bar, 100 μm. One technical replicate of three biological replicates. Unpaired *t*-test, *t*_4_ = 12.99. **b**, *Serpinb2*^+^ neuron ablation decreased 3 h total food consumption by mice. *n* = 8 mice per group. Unpaired *t*-test, *t*_14_ = 5.843. **c**, *Serpinb2*^+^ neuron ablation has a long-term effect on bodyweight (BW) loss. *n* = 9 mice per group. Two-way ANOVA, Šídák’s test (left), *F*_1,16_ = 15.78; unpaired *t*-test (right), *t*_16_ = 5.428. **d**, Oxygen consumed (VO_2_) by GFP-expressing control (*n* = 8) and taCasp3 (*n* = 8) mice fed with chow. Each symbol represents the mean of eight individual mice (left); data are presented as mean ± s.e.m. (right). Unpaired *t*-test, *t*_14_ = 3.692 (light), *t*_14_ = 5.354 (dark). **e**, Carbon dioxide consumed (VCO_2_) by GFP-expressing control (*n* = 8) and taCasp3 (*n* = 8) mice fed with chow. Each symbol represents the mean of eight individual mice (left); data are presented as mean ± s.e.m. (right). Unpaired *t*-test, *t*_14_ = 2.575 (light), *t*_14_ = 3.799 (dark). **f**, Energy expenditure of mice by GFP-expressing control (*n* = 8) and taCasp3 (*n* = 8) mice fed with chow. Each symbol represents the mean of eight individual mice (left); data are presented as mean ± s.e.m. (right). Unpaired *t*-test, *t*_14_ = 3.462 (light), *t*_14_ = 5.028 (dark). **g**, Cumulative food consumption from baseline to day 3. GFP-expressing control (*n* = 7) and taCasp3 (*n* = 7) mice fed with chow. Each symbol represents the mean of seven individual mice (left); data are presented as mean ± s.e.m. (middle, right). Two-tailed, unpaired *t*-test, *t*_12_ = 4.969 (middle), *t*_12_ = 0.4510 (light), *t*_12_ = 3.534 (dark). Source data

**Extended Data Fig. 7:**
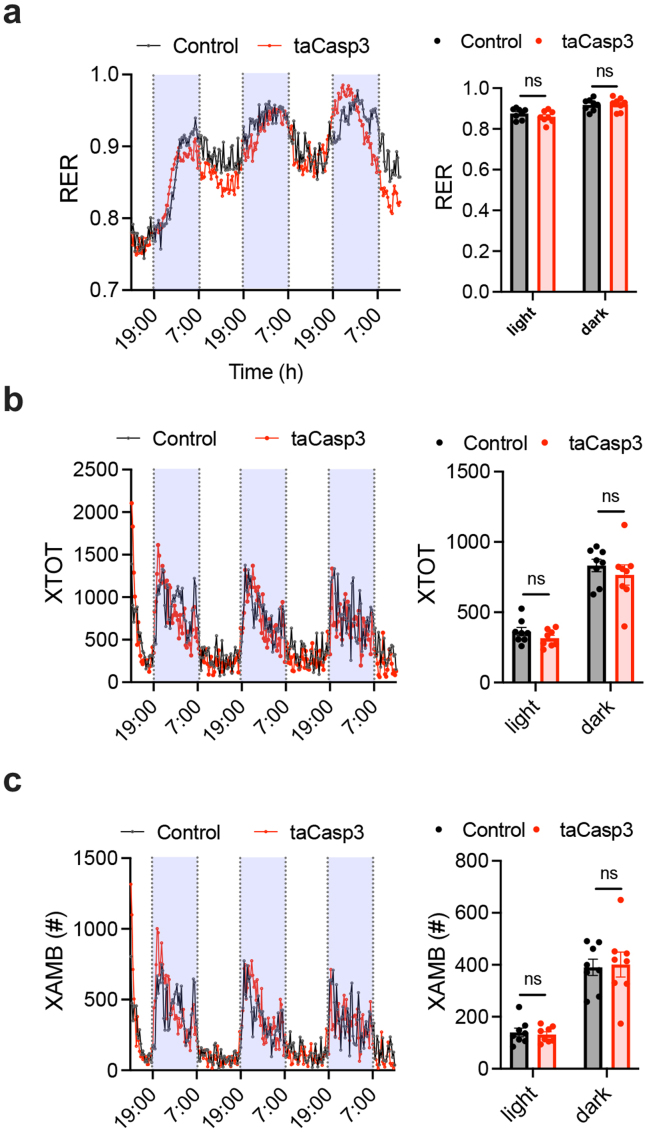
Ablation of *Serpinb2*^+^ neurons does not affect RER and locomotion. **a**, Respiratory exchange ratio (RER) of mice GFP-expressing control (n = 8) and taCasp3 (n = 8) mice fed chow. Two-tailed, unpaired t-test, t_14_ = 1.162 (light), t_14_ = 0.1953 (dark). **b**, Total activity, GFP-expressing control (n = 8) and taCasp3 (n = 8), two-tailed, unpaired t-test, t_14_ = 1.297 (light), t_14_ = 0.7952 (dark). **c**, Ambulatory movement, GFP-expressing control (n = 8) and taCasp3 (n = 8), two-tailed, unpaired t-test, t_14_ = 0.4021 (light), t_14_ = 0.1843 (dark). Data are presented as means ± SEM. Source data

To assess whether the loss of function of the *Serpinb2*^+^ neurons can exert a long-term effect on energy homeostasis, we selectively ablated NAcSh *Serpinb2*^+^ neurons in *Serpinb2-*Cre mice by injecting a Cre-dependent AAV vector expressing caspase-3, which eliminates the neurons by inducing cell death (Fig. 6a). Ablation of the NAcSh *Serpinb2*^+^ neurons resulted in decreased food intake (Fig. 6b) as well as reduced bodyweight gain (~10% or 2 g over 7 weeks) despite food and water being freely available in the home cage (Fig. 6c). A reduction in bodyweight is normally caused by an imbalance between energy intake and expenditure. To this end, we analyzed the effect of *Serpinb2*^+^ neuron ablation on metabolism by performing metabolism recording, which measures the volumes of oxygen consumption (VO_2_), carbon dioxide production (VCO_2_), respiratory exchange ratio (RER), energy expenditure, total movement, ambulatory movement and food intake (Fig. 6d–g and Extended Data Fig. 7a–c). We found that *Serpinb2*^+^ neuron ablation (the taCasp3 group) significantly increased VO_2_ consumption (Fig. 6d), VCO_2_ production (Fig. 6e), energy expenditure (Fig. 6f) as well as decreasing cumulative food intake (Fig. 6g). However, no significant change in RER or movement was observed (Extended Data Fig. 7a–c). Given that a previous study indicated that the NAcSh-to-LH pathway could modulate energy expenditure^58^, the metabolic changes observed could be the result of a lack of innervation from *Serpinb2*^+^ neurons. Collectively, these results demonstrate that ablation of *Serpinb2*^+^ neurons reduces long-term bodyweight gain, not only by reducing food intake but also by increasing energy expenditure.

## Discussion

Feeding is an essential goal-directed behavior that is heavily influenced by homeostatic state and motivation. The accumbal-to-lateral hypothalamic pathway has been implicated in regulating feeding behavior, but the specific neuron subtypes and precise neuronal circuits in the LH are not clear. In this study, we filled in this knowledge gap by identifying a NAcSh D1 neuronal subtype and the associated circuit that integrates neuronal and humoral signals to regulate food consumption. Specifically, we identified a *Serpinb2-*expressing D1-MSNs subtype located in the NAcSh that regulates feeding behavior through the projection to LH^LepR^ neurons, providing innervations to LH^LepR^ neurons from outside the hypothalamus. We demonstrate that the *Serpinb2*^+^ neurons bidirectionally modulate food motivation and consumption specifically without affecting other motivated behaviors, such as cocaine CPP, sucrose preference or social interaction. Importantly, *Serpinb2*^+^ neurons target the LepR-expressing GABAergic neurons in the LH, and their activation can partly overcome the suppressive effect of leptin on food intaking, whereas their ablation leads to increased energy expenditure and decreased food consumption, cumulating in bodyweight loss.

### The *Serpinb2*^+^ neurons are functionally distinct from the pan D1-MSNs in NAcSh

Previous studies have observed reduced activity of D1-MSNs during food consumption, and, consistently, suppressing D1-MSNs activity prolonged food intake^22^. Using *Serpinb2*-Cre, *Tac2*-Cre and *Drd1*-Cre mice, we compared the *Serpinb2*^+^, *Tac2*^+^ and *Drd1*^+^ MSNs in regulating feeding behaviors and found that manipulations of these neuron subtypes led to different outcomes (Figs. 2 and 3). First, *Serpinb2*^+^ neurons are activated by food consumption but suppressed after the end of eating, whereas *Drd1*^*+*^ neurons are activated during the approach to food and after the end of eating. Second, *Serpinb2*^+^ neurons bidirectionally regulate food seeking and intake, whereas manipulating *Drd1*^*+*^ neurons does not significantly alter feeding behavior. On the other hand, a previous study showed that inhibition of *Drd1*^*+*^ neurons promoted liquid fat food intake^22^. This discrepancy might be because of the different feeding assays used in the two studies. We used home cage free-access food intake in this study, whereas the previous study used head-fixed mice licking liquid fat food as the assay^22^. Third, ablation of the *Serpinb2*^+^ neurons significantly reduced food intake (Fig. 6), which is consistent with our finding that *Serpinb2*^+^ neuron activation positively regulates food intake (Fig. 3). However, a previous study indicated that lesions or inactivation of the NAc neurons do not significantly alter food consumption^59^. We do not consider these results to be in conflict, as NAc is composed of many D1-MSN and D2-MSN neuron subtypes, of which many may not be involved in regulating food intake, while others can positively or negatively regulate food intake. Consequently, manipulating *Serpinb2*^+^ neurons versus the entire NAc neurons can have different outcomes. Indeed, using a genetically engineered Cre-off virus, we demonstrated that *Serpinb2*^+^ neurons and *Serpinb2*^−^ neurons have opposite roles in regulating feeding (Fig. 3c,d compared to Fig. 3f,g). Our results indicate that finer granularity and cell type-specific approaches are needed to dissect the functions of different neuron subtypes in the NAc. For example, although *D2*^+^ neuronal activity is not altered during food consumption^22,30^, D2 receptors are indeed downregulated in individuals with obesity^60,61^. Whether certain D2-MSN subtypes are involved in regulating food intake remains to be determined.

### The NAcSh^*Serpinb2*+^–LH^*LepR*+^ circuit controls feeding in the hungry state

It has been reported that either LH GABA or Vglut2 neuronal subpopulation can receive NAc innervation^22,62^. However, the LH GABA and Vglut2 neurons are highly heterogeneous and can be further divided into 15 distinct populations, respectively^63^. Thus, the specific cell types that receive NAc innervation were unknown. Previous studies also showed that NAcSh D1-MSNs to LH inhibitory transmission stops eating, and endocannabinoids-mediated suppression of this projection promotes excessive eating of highly palatable chow^26^, but the D1 subtype involved in this projection was not known. Using viral tracing, we discovered that the NAcSh *Serpinb2*^+^ D1-MSNs project to *LepR*^+^ neurons in the LH underlying the *Serpinb2*^+^ neuron function in food intake (Fig. 5e–j). Distributed in numerous regions involved in the regulation of energy balance, the *LepR*^+^ neurons lie in the mediobasal hypothalamic 'satiety centers' and in the LH, which is regarded as the 'feeding center'^54,64^. Leptin treatment induced *cFos* expression, and 100 nM of leptin depolarized 34% of LepR-expressing neurons in the LH^21^. Unilateral intra-LH leptin decreased food intake and body weight^21^. In our study, we found that activation of the *Serpinb2*^+^ neurons increased inhibition of *LepR*^+^ neuron excitability, resulting in increased food consumption; whereas inhibition of *Serpinb2*^+^ neurons decreased the inhibition of *LepR*^+^ neuron excitability, leading to decreased food consumption even after fasting (Figs. 3d and 4h). Our results are consistent with previous reports demonstrating that LH LepR neuron activation decreases chow intake^65^. Importantly, manipulating *Serpinb2*^+^ neuronal activity could at least partly override the effect of leptin in the LH to modulate food consumption (Fig. 5m). Our study thus reveals a parallel and compensatory circuit from NAcSh to LH^LepR^ in addition to the hypothalamus circuit that directly modulates food intake to maintain energy homeostasis.

### Neuronal circuits regulate feeding behavior and metabolism beyond the hypothalamus

Neurons within the central nervous system receive humoral and central signals that ultimately regulate eating behavior and metabolism. Earlier studies suggested that the dorsal and lateral hypothalamic areas of the hypothalamus were the feeding center. Recent works have connected feeding behavior regulation to the NAcSh-to-LH projection. In our study, we conducted an operant food intake assay and found that *Serpinb2*^+^ neuronal activity bidirectionally regulates active lever presses and the earned reward, which reflects appetitive food motivation (Fig. 3i,j). The role of *Serpinb2*^+^ neurons in regulating energy homeostasis and appetitive motivation is consistent with previous findings that the NAc is involved in integrating descending signals pertaining to homeostatic needs and goal-related behaviors^8,66^. On the other hand, ablation of the *Serpinb2*^+^ neurons leads to bodyweight loss, which is a combined effect of reduced food intake and increased energy expenditure. These observations raise the possibility that the NAcSh^Serpinb2+^-to-LH^LepR+^ projection may provide a regulatory mechanism that enables rapid switching between different behavioral states in response to changing external conditions. Lack of the *Serpinb2*^+^ neuronal function disrupts energy homeostasis and results in long-term bodyweight loss. Clinically, activation of the NAcSh *Serpinb2*^+^ neurons may provide a potential treatment strategy for anorexia or obesity.

In conclusion, by focusing on the neuron subtypes located in the NAcSh, we identified a molecularly defined neuron subtype that can regulate food intake through a neuron–hormone axis. Our detailed characterization of the *Serpinb2*^+^ neuron subtype not only clarified previous discrepancies regarding the role of the NAcSh-to-LH circuit in regulating feeding behavior but also revealed *LepR*^+^ neurons in the LH as the downstream neurons receiving the inputs, thus linking neuronal and hormonal regulation. In addition, our findings have clinical implications, as activating or ablating the *Serpinb2*^+^ neurons in NAcSh could regulate food intake and metabolism. Thus, the NAcSh *Serpinb2*^*+*^ neurons could be an ideal entry point for understanding the complex brain–metabolism regulatory network underlying eating and bodyweight control.

## Methods

### Animals

All experiments were conducted in accordance with the National Institute of Health Guide for Care and Use of Laboratory Animals and approved by the Institutional Animal Care and Use Committee (IACUC) of Boston Children’s Hospital and Harvard Medical School (protocol number IS00000270-6). The *Serpinb2*-Cre mice were generated by Cyagen USA. The *Tac2*-Cre knock-in mouse line was a gift from Q. Ma at Dana-Farber Cancer Institute and Harvard Medical School. The 129-Tg (Drd1-Cre)120Mxu/Mmjax mice (Jax, 037156), B6.Cg-Gt(ROSA)26Sor^tm9(CAG-tdTomato)Hze^/J (Jax, 007909) and C57BL/6NJ (Jax, 000664) mice were purchased from Jackson lab. For behavioral assays, 12–16-week-old male mice were used unless otherwise specified. The mice were housed in groups (3–5 mice per cage) in a 12 h light/dark cycle (light time, 07:00–19:00 h), with food and water provided ad libitum unless otherwise specified. Ambient temperature (23–25 °C) and humidity (55–62%) were automatically controlled.

### FISH and immunofluorescence staining

Mice were transcardially perfused with PBS followed by 4% paraformaldehyde. Brains were then placed in a 30% sucrose solution for 2 days. The brains were frozen in optimal cutting temperature (OCT) embedding media and 16 μm (for FISH) or 35 μm (for immunofluorescence) coronal sections were cut with a vibratome (Leica, no. CM3050 S). For FISH experiments, the slices were mounted on SuperFrost Plus slides and air dried. The multi-color FISH experiments were performed following the manufacturer's instructions for the RNAscope Fluorescent Multiplex Assay (ACD Bioscience). The probes used in this study were Tac2 (ACD, cat. no. 446391-C2), Serpinb2 (cat. no. 538091), Drd1 (cat. no. 461901-C3), Drd2 (cat. no. 406501-C3), Spon1 (cat. no. 492671), Stard5 (cat. no. 880931-C3) and Lepr (cat. no. 402731-C3). For immunofluorescence, cryostat sections were collected and incubated overnight with blocking solution (1× PBS containing 5% goat serum, 5% BSA and 0.1% Triton X-100) and then incubated with the following primary antibodies, diluted with blocking solution, for 1 day at 4 °C: rabbit anti-cFos (1:2,000; Synaptic systems, cat. no. 226003), chicken anti-GFP (1:2,000; Aves Labs, cat. no. GFP-1010), chicken anti-mCherry (1:2,000; Novus Biologicals, cat. no. NBP2-25158), mouse anti-orexin-A (KK09) (1:500; Santa Cruz Biotechnology, cat. no. sc-80263), rabbit anti-GABA (1:1,000; Sigma, cat. no. A2052), rabbit anti-MCH (1:20,000; Phoenix Pharmaceuticals, cat. no. H-070-47) and rabbit anti-leptin receptor (1:1,000; Abcam, cat. no. 104403). Samples were then washed three times with washing buffer (1× PBS containing 0.1% Tween-20) and incubated with the Alexa Fluor conjugated secondary antibodies (Alexa Fluor 488 goat anti-chicken IgY (H+L) (1:500; Invitrogen, cat. no. A11039, lot no. 2304258); Alexa Fluor 488 donkey anti-rabbit IgG (H+L) (1:500; Invitrogen, cat. no. A32790, lot no. WF320931); Alexa Fluor 568 donkey anti-rabbit IgG (H+L) (1:500; Invitrogen, cat. no. A10042, lot no. 2306809)) for 2 h at room temperature. The sections were mounted and imaged using a Zeiss LSM800 confocal microscope with EC plan-Neofluar 10×/0.30 M27 or Plan-Apochromat 20×/0.8 M27 objectives or an Olympus VS120 Slide Scanning System.

### AAV vectors

The following AAV vectors (with a titer of >10^12^) were purchased from UNC Vector Core: AAV5-EF1a-DIO-hChR2(H134R)-EYFP, AAV5-EF1a-DIO-EYFP and AAV-DJ-EF1a­DIO­GCaMP7s. The following AAV vectors were purchased from Addgene: AAV5-hSyn-DIO-hM3D(Gq)-mCherry (cat. no. 44361), AAV5-hSyn-DIO-hM4D(Gi)-mCherry (cat. no. 44362), AAV5-hSyn-DIO-mCherry (cat. no. 50459), pAAV-flex-taCasp3-TEVp (cat. no. 45580), pAAV-Ef1α-DIO-eNpHR 3.0-EYFP (cat. no. 26966) and pAAV-hSyn-FLEx-mGFP-2A-synaptophysin-mRuby (cat. no. 71760). The following AAV vectors were purchased from BrainVTA: rAAV-hSyn-DO-hM3D(Gq)-EGFP-WPREs (PT-2155), rAAV-hSyn-DO-hM4D(Gi)-EGFP-WPREs (PT-6701), rAAV-hSyn-DO-EGFP-WPREs (NA), rAAV-Ef1a-DIO-mCherry-F2A-TVA-WPRE-hGH-polyA (PT0023), rAAV-EF1a-DIO-RVG (PT0207) and RV-ENVA-ΔG-EGFP (R01001).

### Stereotaxic brain surgeries

The injection was performed using a small-animal stereotaxic instrument (David Kopf Instruments, model 940) under general anesthesia by isoflurane (0.8 l min^−1^; isoflurane concentration 1.5%) in oxygen. A feedback heater was used to keep mice warm during surgeries. Once the mouse skull was exposed, a cranial window (1–2 mm^2^) was drilled unilaterally (for in vivo photometry, monosynaptic rabies and retrograde tracing experiments) or bilaterally (for optogenetic and chemogenetic experiments). Next, a glass capillary was lowered into the window to deliver approximately 0.1–0.15 μl of AAV vector to the area of interest. The viral solution was delivered at a rate of 1 nl s^−1^ using a nanoliter injector (Nanoject III, Drummond Scientific 3000207). Following delivery, the pipette was left in place for 10 min and carefully withdrawn. For in vivo photometry and optogenetics experiments, following viral injection, a fiber optic cannula (200 μm in diameter; Inper Inc.) was implanted 0.1 mm above the viral injection site and secured with dental cement (Parkell, cat. no. S380). For the drug delivery cannula implantation, the cannula (guide cannula, CC 2.0 mm, C = 4.5 mm; injector: G1 = 0.5 mm; dummy cannula: G2 = 0; RWD Life Science) was directly implanted 0.5 mm above the LH and secured with dental cement (Parkell, cat. no. S380). The coordinates of viral injection and implantation sites are based on previous literature and The Mouse Brain in Stereotaxic Coordinates (third edition) as follows: NAc (anterior–posterior (AP), +1.2 mm; medial–lateral (ML), ±0.6 mm; dorsal–ventral (DV), −4.5 mm) and LH (AP, −1.3 mm; ML, ±1.2 mm; DV, −5.0 mm). Mice were allowed to recover in a warm blanket before they were transferred to housing cages for 2–4 weeks before behavioral evaluation was performed.

### Neuronal tracing

For CTB tracing, mice were injected with 0.1–0.2 μl CTB-555 (AF-CTB, all from Life Technologies) unilaterally into the LH (AP, −1.3 mm; ML, +1.2 mm; DV −5 mm). To identify where *Serpinb2*^+^ neurons form synapses, *Serpinb2*-Cre mice were unilaterally injected with 0.1–0.15 μl pAAV-hSyn-FLEx-mGFP-2A-synaptophysin-mRuby in the NAcSh. Then, 10 days after CTB injections and 3 weeks after virus injections, brain tissue was collected and processed for confocal imaging. Mice used for rabies tracing were first unilaterally injected with the starter AAV. After 14 days, the same mice were injected with the rabies virus; 7 days later, the mice were killed and their brain tissue was collected and processed for FISH. To aid in visualization, images were adjusted for brightness and contrast using ImageJ2 (version 2.9.0/1.53t), but alterations always were applied to the entire image.

### Fiber photometry during feeding

The *Serpinb2*^+^, *Drd1*^+^ and *Tac2*^+^ neuronal dynamics during feeding were measured using fiber photometry. Following injection of an AAV1-hSyn-FLEX-GCaMP7s vector into NAcSh of *Serpinb2*-Cre, *D1*-Cre and *Tac2*-Cre mice, an optical cannula (Ø200 μm core, 0.37 numerical aperture) was implanted 100 μm above the viral injection site. Mice were allowed to recover for 3 weeks and then subjected to behavioral testing. GCaMP fluorochrome was excited when GCaMP-expressing neurons were excited, and emission fluorescence was acquired with the RZ10X fiber photometry system, which has built-in LED drivers, LEDs and photosensors (Tucker–Davis Technologies). The LEDs include 405 nm (as isosbestic control) and 465 nm (for GCaMP excitation). Emitted light was received through the Mini Cube (Doric Lenses) and split into two bands: 420–450 nm (autofluorescence) and 500–550 nm (GCaMP7 signal). Mice with an optical cannula were attached to recording optic cables, and the LED power at the tip of the optical cables was adjusted to the lowest possible setting (~20 μW) to minimize bleaching. Mice behaviors were recorded by CCD cameras (SuperCircuits).

For the home cage food consumption recording, fasted mice were habituated to the fiber optic cord for 10 min in the home cage with normal bedding. After that, we put two pieces of chow (PicoLab Diet, 5053) and one Lego block on the bedding on opposite sides of the cage. During this 10 min phase, mice can sense the food and freely eat. Behavioral events, such as baselines of free moving, start eating, end eating, sniffing or leaving the Lego block were scored manually and synchronized with the fluorescence signal based on recorded videos. The voltage signal data stream was acquired with Synapse software (Tucker–Davis Technologies, version 44132) and exported, filtered and analyzed with Matlab code provided by TDT offline data analysis tools (https://www.tdt.com/docs/sdk/offline-data-analysis/offline-data-matlab/fiber-photometry-epoch-averaging-example). We segmented the data based on individual trials of different events. To calculate Δ*F*/*F*, a polynomial linear fitting was applied to the isosbestic signal to align it to the GCaMP7 signal, producing a fitted isosbestic signal that was used to normalize the GCaMP7 as follows: Δ*F*/*F* = (GCaMP7 signal − fitted isosbestic) / fitted isosbestic signal. The *z*-score of Δ*F*/*F* of the heat map was then calculated as:

$${z\rm{-score}}=\frac{{\rm{V}}_{{\rm{signal}}}-\overline{{\rm{V}}_{{\rm{basal}}}}}{{\rm{STD}}({\rm{V}}_{{\rm{basal}}})}$$

For the Ca^2+^ traces and heat map, multiple trials were averaged for each mouse. Each data point presented represents one individual mouse recorded. Data are presented using the mean and standard deviation of the signal during the baseline periods (the pooled 10 s time windows before each stimulus). ΔPeak (Δ*F*/*F*) was measured by subtracting the peak Δ*F*/*F* (−10 s, 0 s) from the peak Δ*F*/*F* (0 s, 10 s). Time 0 s indicates the start time point of each event. Power analysis was performed using the G*Power 3 program^67^, with parameters including a two-tailed paired *t*-test and a significance level of α = 0.05; the power of this experiment was >0.95.

### Behavioral assays

#### Open field tests

A clear box (27.3 cm × 27.3 cm square base with 20.3 cm high walls) was used for the open field test, and the center zone was 36% of the total area. Before testing, mice were habituated to the test room for at least 20 min. Mice were placed in the center of the box at the start of the assay. Movements were recorded (Med Associates, ENV-510) for 1 h in 5 min bins. In addition to regular parameters related to locomotor activity (such as total travel distance, velocity, ambulatory time, resting time), time spent and distance traveled in the center area of the testing arena were also recorded and analyzed.

#### CPP

Mice were allowed to freely explore both sides of a custom-made (Med Associates) CPP training apparatus (L × D × H, 25 × 19 × 17 cm) for 30 min. Trajectories were tracked by infrared photobeam detectors, and the travel distance and duration were recorded (Med Associates, ENV-510) to assess their baseline place preference. Mice that showed strong bias (>75% preference) were excluded from the experiments. Then, for chemogenetic activation or inhibition during CPP formation, the mice were intraperitoneally injected with saline and confined to their preferred side of the chamber for 30 min before being returned to their home cage. At least 4 h later, the same mice received CNO (Cayman Chemical Company, item no.16882) at least 15 min before being confined to their non-preferred side of the chamber for 30 min. They were then returned to their home cage. The same training with saline and CNO injection was performed for three consecutive days. Then, 24 h after the final training session, mice were re-exposed to the CPP chamber and allowed to explore both sides of the chamber for 30 min. For the cocaine CPP, mice received CNO at least 15 min before intraperitoneal injection of 15 mg kg^−1^ cocaine and then were confined to their non-preferred side of the chamber for 30 min during conditioning days.

#### Sucrose preference test

Mice were singly housed for at least 2 days before the tests. For the first 2 days, animals were habituated with two bottles of water, with the position of the two bottles switched at 24 h. On the night of day 2, mice were water-deprived for 16 h and then the test was started on day 3. The test period lasted for 3 h, during which the mice were exposed to one bottle of pure drinking water and one bottle of drinking water containing 2% sucrose solution (w:v). Bottles were weighed before and after the tests to measure the water consumption. The sucrose preference ratio was calculated by dividing the consumption of sucrose solution by the total consumption of both pure drinking water and sucrose solution.

#### Three-chamber social interaction

Each social interaction chamber (30 × 30 × 30 cm) contained dividing walls with an open middle section to allow access. Both outer chambers contained wire cups. Mice were given free access to the apparatus for 10 min (in the absence of other mice) to habituate and confirm initial unbiased preference. The time spent in each chamber was recorded, and the time spent in close interaction with the nose point within 2 cm of the enclosure was also recorded (EthoVision XT 14, Noldus Information Technology). To test for sociability, mice were placed into the middle chamber of the apparatus with one outer chamber containing one mouse ('stranger 1') confined in a wire cup and the other chamber containing a Lego block. For social novelty preference, mice were again placed into the middle chamber with one chamber containing the familiar mouse (stranger 1) and the other containing a novel mouse ('stranger 2') confined in a wire cup. Male familiar and novel mice introduced for assay in social interactions matched the male test subject. For each phase, the test mice explored the entire arena throughout the 10-min trial. The time spent interacting with the empty wire, stranger 1 and stranger 2 mice during the 10-min session was recorded^68^.

#### Elevated plus maze

An elevated plus maze (EPM) was used to measure anxiety effects. Before the EPM test, mice were brought to the testing room for environmental habituation for at least 30 min. The EPM apparatus consisted of an elevated platform (80 cm above the floor) with four arms (each 30 cm in length and 5 cm in width), two opposing closed arms with 14 cm walls and two opposing open arms. Mice were individually placed in the center of the EPM apparatus, towards one of the open arms. The trajectories of the mice were tracked for 5 min, and the time spent in the open arms was recorded with an Ethovision video tracking system V14 (Noldus Information Technology).

#### Post-fasted food intake

Mice were individually placed in the home cage and fasted overnight (18 h), achieving 90% of their original body weight. Mice that were over 92% and lower than 88% of their original body weight were excluded from the test. Mice received CNO (2 mg ml^−1^ for the hM3Dq group and 5 mg ml^−1^ for the hM4Di group) by intraperitoneal injection and then regular chow pellets (3 g per pellet; PicoLab Diet, 5053) were put in the hopper. The remaining food pellets were collected 3 h later and measured to calculate the total amount of food consumed (g). For the leptin titration test, 100 ng, 500 ng and 1 μg of leptin (R&D, cat. no. 498-OB) or the same volume of saline was delivered through the cannula by pump to the LH area for 5 min; after 5 min, the pellets were added. For the leptin treatment test on DREADDs mice, 15 min after CNO injection, 1 μg or 300 ng of leptin was delivered. For the taCasp3 group, the test was carried out 3 weeks after taCasp3 virus injection.

#### Food-approach test

Animals were placed in a custom three-chamber arena (45 × 60 × 35 cm) to assess the time spent in a designated food zone area. The arena contained two 64 cm^2^ food cups in two outer corners of separate chambers. One cup contained standard grain-based chow (Harlan) and the other cup contained one Lego block. Mice were allowed to explore the arena freely, and spatial locations were tracked using the Ethovision video tracking system V14 (Noldus Information Technology) and CCD cameras (SuperCircuits). The proportion of time spent in the food zone was calculated as the time spent in the food zone divided by the total time spent in both the food and non-food zones.

#### Operant behavior

Mice were individually placed in the home cage and fasted to maintain 90% of their original body weight. Mice over 92% and lower than 88% of their original body weight were excluded from the test. Animals were first given access to 20 mg sweetened chow pellets daily (Bio-Serv, 0071) in their home cage before testing and then trained to enter the operant chamber (Med Associates) to retrieve a pellet. Each pellet was delivered 10 s after the prior pellet retrieval. After at least 2 days of training and until >30 pellets were earned in a single session, animals were trained for the fixed ratio 1 task, in which each act of pressing a lever was rewarded with one pellet. A new trial did not begin until the animals entered the magazine to retrieve a pellet. Retrieval was followed by a 5 s intertrial interval, after which the levers were reactivated, indicated by a cue light. Training continued until >40 pellets were earned in a single 60 min session.

#### Progressive ratio

After the fixed ratio 1, 3 and 5 training sessions, all mice were tested with CNO treatment. For the progressive ratio task, a schedule of reinforcement was used in which each subsequent reward required exponentially more lever pressing based on the increment formula *v*+ (*n* – 1) × *c*, rounded to the nearest integer, where *v* is the progressive ratio, *n* is the trial number and *c* is the increment parameter^69^. Each session lasted 60 min. Data were collected using the MazeEngineers behavioral software (Conduct Science).

#### Optogenetic modulations of post-fasted food intake

Mice received 20 min of laser stimulation (4 × 5 min; on–off–on–off), after which the remaining food pellets (PicoLab Diet, 5053) were collected and food intake was measured. For photostimulating ChR2, a 473 nm laser (OEM Lasers/OptoEngine) was used to generate laser pulses (10–15 mW at the tip of the fiber, 5 ms, 20 Hz), controlled by a waveform generator (Keysight). The total duration of the behavioral test was 20 min, which was divided into four 4 × 5 min epochs (with the laser on, off, on and off, respectively). For NpHR photostimulation, a 532 nm laser (OEM Lasers/OptoEngine) generated constant light of 8–10 mW power at each fiber tip.

#### CLAMS recording

Mice were housed singly and habituated to the metabolic cages (CLAMS, Columbus) for at least 3 days before testing under a 12 h light/12 h dark cycle. Mice used in the control and experimental groups (that is, GFP and taCasp3 mice) were age-matched. Locomotor activity (infrared beam breaks in the *x*, *y* and *z* axis), energy expenditure, VO_2_, VCO_2_, RER and food intake were recorded. Data were exported using Clax software v.2.2.0 and were analyzed in Prism 9. White and purple represent light (07:00–19:00 h) and dark (19:00–07:00 h) cycles, respectively. Energy expenditure, VO_2_ and VCO_2_ data were normalized to body weight. The mice were fed with regular chow (PicoLab rodent diet 20, 5053; physiological value, 3.43 kcal g^–1^). Food and water were freely available during testing. Gas sensor calibration (CO_2_ and O_2_) of the apparatus was performed before each test. Mouse body weight was recorded before and after every testing session.

### Statistics

All statistical analyses were performed using GraphPad Prism (version 9) software and fiber photometry results were analyzed by MATLAB. No statistical methods were used to pre-determine sample sizes but our sample sizes are similar to those reported in previous publications^70–72^. All mice were randomly assigned to different groups and data collection was randomized whenever possible. Mice that, after histological inspection, had the location of the viral injection (reporter protein), cannula implantation, or the optic fiber(s) outside the area of interest were excluded. Data collection and analysis were not performed blind to the conditions of the experiments. Most behavioral experiments were controlled by an automated computer system, and the data were collected and analyzed in an unbiased way. Statistical analyses were two-tailed. Parametric tests including paired and unpaired *t*-test and one-way ANOVA were used if distributions passed the Kolmogorov–Smirnov normality test. Normality tests were not performed for one-way ANOVA with missing values. If data were not normally distributed, non-parametric tests were used. One-sample *t*-test was performed to determine whether the group mean differed from a specific value. For comparisons across more than two groups, one-way ANOVA or repeated-measures one-way ANOVA was performed for normally distributed data, followed by Tukey’s multiple comparison tests; two-way ANOVA was performed for differences between groups with two independent variables, followed by Šídák’s multiple comparison tests.

All significant *P* values (*P* < 0.05) are indicated in the figures. For detailed statistical analysis, see the figure legend with each figure.

### Reporting summary

Further information on research design is available in the Nature Portfolio Reporting Summary linked to this article.

## Supplementary information

Reporting Summary

## Source data

Source Data Fig. 1 Statistical source data.

Source Data Fig. 2 Statistical source data.

Source Data Fig. 3 Statistical source data.

Source Data Fig. 4 Statistical source data.

Source Data Fig. 5 Statistical source data.

Source Data Fig. 6 Statistical source data.

Source Data Extended Data Fig./Table 1 Statistical source data.

Source Data Extended Data Fig./Table 2 Statistical source data.

Source Data Extended Data Fig./Table 3 Statistical source data.

Source Data Extended Data Fig./Table 4 Statistical source data.

Source Data Extended Data Fig./Table 5 Statistical source data.

Source Data Extended Data Fig./Table 7 Statistical source data.

## Data Availability

The MERFISH data are available at the Brain Image Library (https://download.brainimagelibrary.org/fc/4c/fc4c2570c3711952/). Source data are provided with this paper.

## Code availability

No custom code was used in the study.
