# Publisher Correction: A subset of dopamine receptor-expressing neurons in the nucleus accumbens controls feeding and energy homeostasis

**DOI:** 10.1038/s42255-025-01285-y

**Published:** 2025-04-01

**Authors:** Yiqiong Liu, Ying Wang, Zheng-dong Zhao, Guoguang Xie, Chao Zhang, Renchao Chen, Yi Zhang

**Affiliations:** 1 Howard Hughes Medical Institute, Boston Children’s Hospital, Boston, MA USA; 2 Program in Cellular and Molecular Medicine, Boston Children’s Hospital, Boston, MA USA; 3 Division of Hematology/Oncology, Department of Pediatrics, Boston Children’s Hospital, Boston, MA USA; 4 Department of Genetics, Harvard Medical School, Boston, MA USA; 5 Harvard Stem Cell Institute, Boston, MA USA

**Keywords:** Feeding behaviour, Neural circuits, Neuroendocrinology, Feeding behaviour

Correction to: *Nature Metabolism* 10.1038/s42255-024-01100-0, published online 15 August 2024.

In the version of the article initially published, the graphs in Fig. 6b were inadvertently switched and have now been amended in the HTML and PDF versions of the article, as seen in Fig. 1 below.

**Fig. 1:**
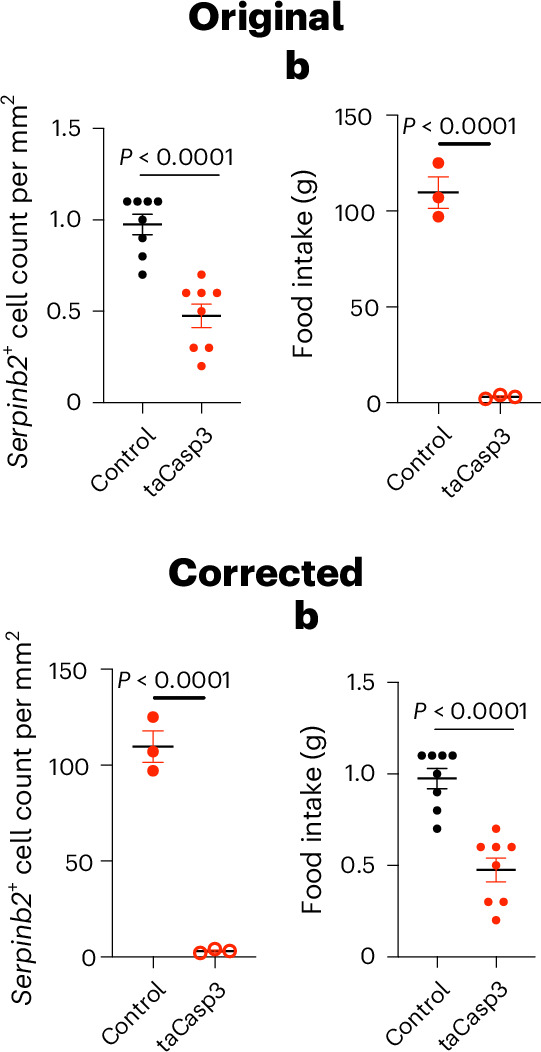
Original and corrected Fig. 6b.

