# Supplementary material for: A subset of dopamine receptor-expressing neurons in the nucleus accumbens controls feeding and energy homeostasis
Source: Nat Metab. 2024 Aug 15;6(8):1616–31. doi: 10.1038/s42255-024-01100-0 (PMC11349581; doi:10.1038/s42255-024-01100-0)
Supplement: Supplementary file 1 — Reporting Summary [file 42255_2024_1100_MOESM1_ESM.pdf]

nature portfolio

Corresponding author(s): Yi Zhang

Last updated by author(s): Jun 16, 2024

Reporting Summary

Nature Portfolio wishes to improve the reproducibility of the work that we publish. This form provides structure for consistency and transparency in reporting. For further information on Nature Portfolio policies, see our Editorial Policies and the Editorial Policy Checklist.

Statistics

For all statistical analyses, confirm that the following items are present in the figure legend, table legend, main text, or Methods section.

n/a	Confirmed	
☐	☒	The exact sample size (*n*) for each experimental group/condition, given as a discrete number and unit of measurement
☐	☒	A statement on whether measurements were taken from distinct samples or whether the same sample was measured repeatedly
☐	☒	The statistical test(s) used AND whether they are one- or two-sided<br>*Only common tests should be described solely by name; describe more complex techniques in the Methods section.*
☒	☐	A description of all covariates tested
☒	☐	A description of any assumptions or corrections, such as tests of normality and adjustment for multiple comparisons
☐	☒	A full description of the statistical parameters including central tendency (e.g. means) or other basic estimates (e.g. regression coefficient) AND variation (e.g. standard deviation) or associated estimates of uncertainty (e.g. confidence intervals)
☐	☒	For null hypothesis testing, the test statistic (e.g. *F*, *t*, *r*) with confidence intervals, effect sizes, degrees of freedom and *P* value noted<br>*Give P values as exact values whenever suitable.*
☒	☐	For Bayesian analysis, information on the choice of priors and Markov chain Monte Carlo settings
☒	☐	For hierarchical and complex designs, identification of the appropriate level for tests and full reporting of outcomes
☒	☐	Estimates of effect sizes (e.g. Cohen's *d*, Pearson's *r*), indicating how they were calculated

Our web collection on statistics for biologists contains articles on many of the points above.

Software and code

Policy information about availability of computer code

Data collection	We listed all softwares used in the experiments and for analysis in the Methods section.<br>For open field test and conditioned place preference test, movement was recorded using Med Associates (Med Associates, St. Albans, VT, ENV-510).<br>For food approaching test, 3 chamber social interaction test and elevated plus maze test, data were collected with Ethovision video tracking system V14 (Noldus Information Technology Inc., Leesburg, VA, USA).<br>For the operant food intake test, mice were trained in a modular operant chamber (Med Associates Inc., St. Albans, VT, USA) and data were collected using MazeEngineers behavioral software (Conduct Science, IL, USA).<br>The fiber photometry signals were acquired with Synapse software (Tucker-Davis Technologies, version 44132)and were exported, filtered, and analyzed with Matlab code provided by TDT offline data analysis tools (https://www.tdt.com/docs/sdk/offline-data-analysis/offline-data-matlab/fiber-photometry-epoch-averaging-example/).<br>For optogenetic stimulation, laser was generated by OEM Lasers/OptoEngine machine.<br>For CLAMS recording, data were exported using Clax software v2.2.0 .<br>IHC and FISH images were collected using a Zeiss LSM800 confocal microscope.
Data analysis	We listed all softwares used in the experiments and the data analysis has been described in the Methods section. Images (including those for FISH and histology) were analyzed using ImageJ (V1.53q). All fiber photometry recording data were exported, filtered and analyzed with Matlab code provided by TDT offline data analysis tools (https://www.tdt.com/docs/sdk/offline-data-analysis/offline-data-matlab/fiber-photometry-epoch-averaging-example/). Statistical analyses were performed and plotted with Matlab or GraphPad Prism software 9.

For manuscripts utilizing custom algorithms or software that are central to the research but not yet described in published literature, software must be made available to editors and reviewers. We strongly encourage code deposition in a community repository (e.g. GitHub). See the Nature Portfolio guidelines for submitting code & software for further information.

Data

Policy information about availability of data

All manuscripts must include a data availability statement. This statement should provide the following information, where applicable:

- Accession codes, unique identifiers, or web links for publicly available datasets
- A description of any restrictions on data availability
- For clinical datasets or third party data, please ensure that the statement adheres to our policy

Source data are provided with this paper. The MERFISH data are available at the Brain Image Library (https://download.brainimagelibrary.org/fc/4c/fc4c2570c3711952/). Source data, extended data, statements of data and code availability are available at https://doi.org/10.1038/s41593-021-00938-x32.

Research involving human participants, their data, or biological material

Policy information about studies with human participants or human data. See also policy information about sex, gender (identity/presentation), and sexual orientation and race, ethnicity and racism.

Reporting on sex and gender	N/A
Reporting on race, ethnicity, or other socially relevant groupings	N/A
Population characteristics	N/A
Recruitment	N/A
Ethics oversight	N/A

Note that full information on the approval of the study protocol must also be provided in the manuscript.

Field-specific reporting

Please select the one below that is the best fit for your research. If you are not sure, read the appropriate sections before making your selection.

☒ Life sciences ☐ Behavioural & social sciences ☐ Ecological, evolutionary & environmental sciences

For a reference copy of the document with all sections, see nature.com/documents/nr-reporting-summary-flat.pdf

Life sciences study design

All studies must disclose on these points even when the disclosure is negative.

Sample size	No statistical methods were used to pre-determine sample sizes, but our sample sizes are similar to those reported in previous publications (PMID: 32679036, 36266470, 35960793).
Data exclusions	Mice that, after histological inspection, had the location of the viral injection (reporter protein), cannula implantation, or of the optic fiber(s) outside the area of interest, were excluded.
Replication	All behavioral experiments were independently repeated at least twice. The representative fluorescent images were chosen from two to four independent experiments. To ensure that results are reproducible, we included in the methods all details relative to softwares, reagents, and protocols. We plotted individual data points and provided source data. Information about the statistical test used is reported in the figure legends.
Randomization	All animals are housed in the same room with ambient temperature and proper light-dark cycles. Animals with similar age and body weights were chosen as experimental subjects. All mice were randomly assigned to different groups.
Blinding	Data collection and analysis were not performed blind to the conditions of the experiments. Most behavioral experiments were controlled by automated computer system and the data were collected and analyzed in an unbiased way.

Reporting for specific materials, systems and methods

We require information from authors about some types of materials, experimental systems and methods used in many studies. Here, indicate whether each material, system or method listed is relevant to your study. If you are not sure if a list item applies to your research, read the appropriate section before selecting a response.

Materials & experimental systems

n/a	Involved in the study	
☐	☒	Antibodies
☒	☐	Eukaryotic cell lines
☒	☐	Palaeontology and archaeology
☐	☒	Animals and other organisms
☒	☐	Clinical data
☒	☐	Dual use research of concern
☒	☐	Plants

Methods

n/a	Involved in the study	
☒	☐	ChIP-seq
☒	☐	Flow cytometry
☒	☐	MRI-based neuroimaging

Antibodies

Antibodies used

Rabbit anti-cFos (1:2000, Synaptic systems, #226003)
Chicken anti-GFP (1:2000, Aves Labs, no. GFP-1010)
Chicken anti-mCherry (1:2000, Novus Biologicals, no. NBP2-25158)
Mouse anti-Orexin-A(KK09) (1:500, Santa Cruz Biotechnology, Cat#sc-80263)
Rabbit anti-GABA (1:1000, Sigma, Cat#A2052)
Rabbit anti-MCH (1:20000, Phoenix Pharmaceuticals, Cat#H-070-47)
Rabbit anti-Leptin receptor (1:1000, Abcam, Cat#104403)
Alexa Fluor 488 goat anti-chicken IgY (H+L) (1:500, Invitrogen, REF #A11039, Lot #2304258)
Alexa Fluor 488 donkey anti-rabbit IgG (H+L) (1:500, Invitrogen, REF #A32790, Lot #WF320931)
Alexa Fluor 568 donkey anti-rabbit IgG (H+L) (1:500, Invitrogen, REF #A10042, Lot #2306809)
DAPI (4',6-diamidino-2-phenylindole, Invitrogen, catalogue number D1306) (0.5 µg/ml in PBS) was used to stain nuclei.

Validation

https://sysy.com/product/226003
https://www.aveslabs.com/products/anti-green-fluorescent-protein-antibody-gfp?variant=25144111169636
https://www.novusbio.com/products/mcherry-antibody_nbp2-25158
https://www.scbt.com/p/orexin-a-antibody-kk09
https://www.sigmaaldrich.com/US/en/product/sigma/a2052
https://www.phoenixpeptide.com/products/view/Antibodies/H-070-47
https://www.abcam.com/en-no/products/primary-antibodies/anti-leptin-receptor-antibody-ab104403
https://www.thermofisher.com/antibody/product/Goat-anti-Chicken-IgY-H-L-Secondary-Antibody-Polyclonal/A-11039
https://www.thermofisher.com/antibody/product/Donkey-anti-Rabbit-IgG-H-L-Highly-Cross-Adsorbed-Secondary-Antibody-Polyclonal/A-21206
https://www.thermofisher.com/antibody/product/Donkey-anti-Rabbit-IgG-H-L-Highly-Cross-Adsorbed-Secondary-Antibody-Polyclonal/A10042
https://www.thermofisher.com/order/catalog/product/D1306

Animals and other research organisms

Policy information about studies involving animals; ARRIVE guidelines recommended for reporting animal research, and Sex and Gender in Research

Laboratory animals

The Serpinb2-Cre mice were generated by Cyagen US Inc. The Tac2-Cre knock-in mouse line was a gift from Q. Ma at Dana-Farber Cancer Institute and Harvard Medical School. 129-Tg (Drd1-cre)120Mxu/Mmjax mice (Jax: 037156), B6.Cg-Gt(ROSA)26Sortm9(CAG-tdTomato)Hze/J (Jax:007909) and C57BL/6NJ (Jax:000664) mice were purchased from Jackson lab. For behavioral assays, 12 -16 weeks old male mice were used unless otherwise specified. The mice were housed in groups (3-5 mice/cage) in a 12-hr light/dark cycle (light time, 7:00 to 19:00), with food and water ad libitum unless otherwise specified. Ambient temperature (23-25°C) and humidity (55-62%) were automatically controlled.

Wild animals

The study did not involve wild animals.

Reporting on sex

Behavior tests were performed using male mice.

Field-collected samples

This study did not involve field-collected samples.

Ethics oversight

All experiments were conducted in accordance with the National Institute of Health Guide for Care and Use of Laboratory Animals and approved by the Institutional Animal Care and Use Committee (IACUC) of Boston Children's Hospital and Harvard Medical School (Protocol number: IS00000270-6).

Note that full information on the approval of the study protocol must also be provided in the manuscript.

Plants

Seed stocks	*Report on the source of all seed stocks or other plant material used. If applicable, state the seed stock centre and catalogue number. If plant specimens were collected from the field, describe the collection location, date and sampling procedures.*
Novel plant genotypes	*Describe the methods by which all novel plant genotypes were produced. This includes those generated by transgenic approaches, gene editing, chemical/radiation-based mutagenesis and hybridization. For transgenic lines, describe the transformation method, the number of independent lines analyzed and the generation upon which experiments were performed. For gene-edited lines, describe the editor used, the endogenous sequence targeted for editing, the targeting guide RNA sequence (if applicable) and how the editor was applied.*
Authentication	*Describe any authentication procedures for each seed stock used or novel genotype generated. Describe any experiments used to assess the effect of a mutation and, where applicable, how potential secondary effects (e.g. second site T-DNA insertions, mosiacism, off-target gene editing) were examined.*
